# Discovery and Validation of Molecular Biomarkers for Colorectal Adenomas and Cancer with Application to Blood Testing

**DOI:** 10.1371/journal.pone.0029059

**Published:** 2012-01-19

**Authors:** Lawrence C. LaPointe, Susanne K. Pedersen, Robert Dunne, Glenn S. Brown, Letitia Pimlott, Snigdha Gaur, Aidan McEvoy, Melissa Thomas, David Wattchow, Peter L. Molloy, Graeme P. Young

**Affiliations:** 1 Flinders Centre for Cancer Prevention and Control, Flinders University of South Australia, Adelaide, South Australia, Australia; 2 Clinical Genomics Pty Ltd., Sydney, New South Wales, Australia; 3 Preventative Health National Research Flagship, CSIRO Food and Nutritional Sciences, Sydney, New South Wales, Australia; 4 Preventative Health National Research Flagship, CSIRO Mathematical and Information Sciences, Sydney, New South Wales, Australia; Sun Yat-sen University Cancer Center, China

## Abstract

**Background & Aims:**

Colorectal cancer incidence and deaths are reduced by the detection and removal of early-stage, treatable neoplasia but we lack proven biomarkers sensitive for both cancer and pre-invasive adenomas. The aims of this study were to determine if adenomas and cancers exhibit characteristic patterns of biomarker expression and to explore whether a tissue-discovered (and validated) biomarker is differentially expressed in the plasma of patients with colorectal adenomas or cancer.

**Methods:**

Candidate RNA biomarkers were identified by oligonucleotide microarray analysis of colorectal specimens (222 normal, 29 adenoma, 161 adenocarcinoma and 50 colitis) and validated in a previously untested cohort of 68 colorectal specimens using a custom-designed oligonucleotide microarray. One validated biomarker, *KIAA1199*, was assayed using qRT-PCR on plasma extracted RNA from 20 colonoscopy-confirmed healthy controls, 20 patients with adenoma, and 20 with cancer.

**Results:**

Genome-wide analysis uncovered reproducible gene expression signatures for both adenomas and cancers compared to controls. 386/489 (79%) of the adenoma and 439/529 (83%) of the adenocarcinoma biomarkers were validated in independent tissues. We also identified genes differentially expressed in adenomas compared to cancer. *KIAA1199* was selected for further analysis based on consistent up-regulation in neoplasia, previous studies and its interest as an uncharacterized gene. Plasma *KIAA1199* RNA levels were significantly higher in patients with either cancer or adenoma (31/40) compared to neoplasia-free controls (6/20).

**Conclusions:**

Colorectal neoplasia exhibits characteristic patterns of gene expression. *KIAA1199* is differentially expressed in neoplastic tissues and *KIAA1199* transcripts are more abundant in the plasma of patients with either cancer or adenoma compared to controls.

## Introduction

Colorectal cancer is treatable if detected and removed at an early stage with 95% of patients surviving beyond five years [1]. There is increasing evidence that removing pre-invasive colorectal lesions, i.e. adenomas, by polypectomy lowers the incidence of, and mortality from, colorectal cancer [2]–[4]. Consequently, preventing colorectal cancer by removing screen-detected adenomas is becoming increasingly emphasized as an important aim of colorectal cancer screening. Simple screening tests currently available, however, are suboptimal for adenoma detection, although fecal immunochemical tests for globin are much improved compared to earlier tests [5]–[7]. Population screening programs may be improved if a convenient blood test were available that is sensitive and specific for both the earliest, most treatable stages of colorectal cancer and also sensitive for pre-invasive adenomas. Such a test would facilitate a rational screening approach by allowing us to direct colonoscopy resources to those subjects who are likely to get most benefit from the invasive procedure [8].

Gene expression patterns are increasingly showing promise for identification of candidate biomarkers for colorectal cancer, but these candidates often lack appropriate validation and little data is available for biomarkers that are also sensitive for adenomas. Putative biomarkers resulting from discovery-based research must be rigorously validated if they are to be clinically useful [9]–[12]. Validation, i.e. testing the hypothesis that the candidate biomarkers are genuine indicators of a phenotype, ideally makes use of a patient cohort that is clinically independent of the discovery cohort. Further, correlation between gene expression patterns in tissue and biomarker detection in blood has not been well defined.

The aims of this study were to determine if adenomas and cancers exhibit characteristic patterns of biomarker expression and to explore whether a tissue-discovered (and validated) biomarker is differentially expressed in the plasma of patients with colorectal adenomas or cancer. Particular attention is given to adenoma expression patterns as adenoma biomarkers have largely been ignored in the literature.

We pursued our aim to uncover sensitive biomarkers for both colorectal adenomas and cancer by following a three-phase strategy of discovery, validation and clinical assay testing. First, high-dimensional gene expression microarray data were analysed to discover candidate biomarkers in both cancers and adenomas from the colorectum. To then validate gene expression candidates, a custom-designed oligonucleotide microarray (“Adenoma Biomarker Gene Chip”) was designed and fabricated to contain a broad selection of hypothetical markers found during the discovery phase as well as markers selected from the literature. Candidate biomarkers were validated using the Adenoma Biomarker Gene Chip in an independent set of neoplastic specimens. Lastly, the potential clinical utility of a promising tissue-validated colorectal neoplasia biomarker was measured in RNA extracted from the plasma of colorectal adenoma and cancer patients and colonoscopy-confirmed healthy controls. This sequential process follows the first two stages of a five-stage evaluation of biomarkers proposed by Pepe et al [13].

## Results

### Neoplastic vs. non-neoplastic transcriptome

**Figure 1:**
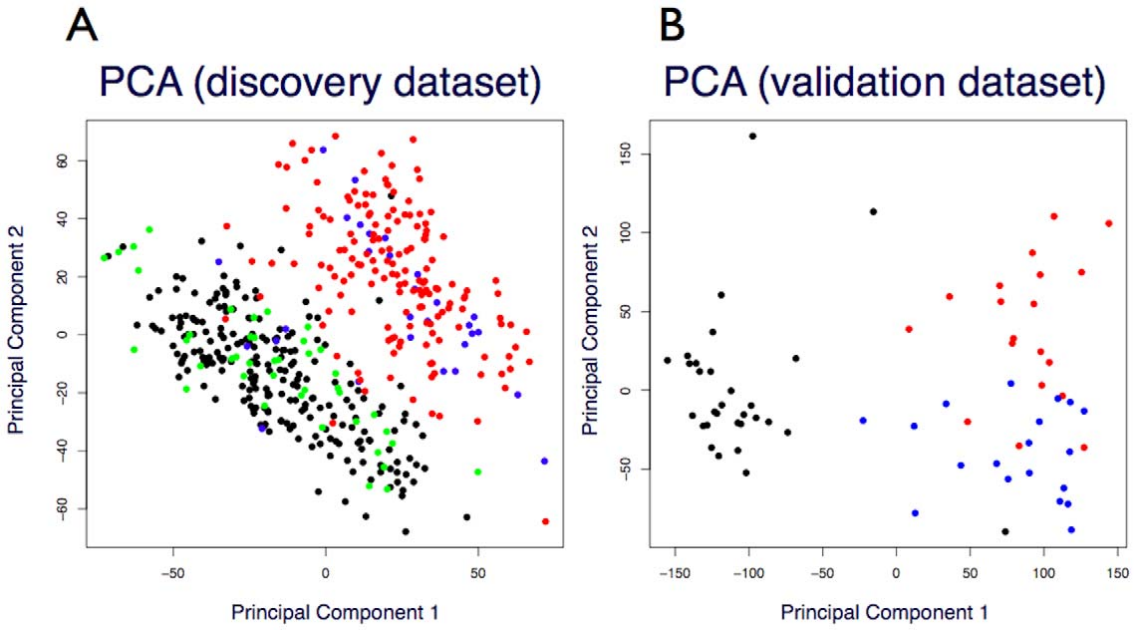
Principal component analysis of microarray gene expression profiles. (A) Discovery microarray dataset: 222 normal, black; 42 colitis/IBD, green; 29 adenomas, blue; and 161 adenocarcinomas, red. (B) Validation microarray dataset: 30 normal, black; 19 adenomas, blue; and 19 adenocarcinomas, red.

Of 44,928 probesets analysed for gene expression difference, we observed 11,183 (24.9%) probesets to be differentially expressed in neoplastic tissues relative to non-neoplastic tissues including colitic specimens. For comparison, we observed 2,701 (6.0%) probesets likewise differentially expressed between normal (n = 222) and colitis (n = 42) tissue extracts (Table 1). These expression data were also analysed at the full genome-level using principal component analysis (PCA) (Figure 1). The largest source of expression change observed in these 454 microarrays (as evidenced by both mean expression change and the PCA plot) correlated with the presence or absence of neoplasia. This phenotypic effect was independent of whether the non-neoplastic tissues exhibited colitis and also independent of whether the neoplastic tissues were adenomatous or cancerous.

**Table 1:** Summary of microarray discovery probesets.

(A) Breakdown of discovered differentially expressed HGU133-A/B Probesets					
Class A	Class B	Diff Exp (p<0.05)	Diff Exp FC>2	Up	Down
Normal & IBD	Adenoma & Cancer	11,183	446	108	338
Normal	Adenoma	3,161	489	106	383
Normal	Cancer	10,897	529	158	371
Normal	IBD	2,701	133	60	73
IBD	Adenoma & Cancer	5,707	527	98	429
IBD	Adenoma	2,561	788	146	642
IBD	Cancer	5,706	537	133	404
Adenoma	Cancer	859	188	145	43

Breakdown of differentially expressed (Diff. Exp) targets for HG-U133-A/B probesets across four phenotypes including normal (222), IBD (42), adenoma (29) and colorectal cancer (161) tissue specimens. Student's t-test and Bonferonni multiple hypothesis test correction (p<0.05) were applied to identify differential expressed probesets. FC>2: Probeset response differs by at least a factor of two. Up: up-regulated probesets in Class B relative to Class A. Down: down-regulated probesets in Class B relative to Class A.

By introducing a requirement for a two-fold change in signal intensity between neoplastic and non-neoplastic tissues, the number of differentially expressed probesets dropped from 11,183 to 446 (Table 1). Thus only 4.0% of the differentially expressed probesets (1.0% of probesets overall) demonstrated at least two-fold expression change. Interestingly, while the number of probesets exhibiting a differential response of any magnitude was approximately equally split between probesets with increased expression in neoplastic tissues (6,227; 55%) and probesets with decreased expression (4,956; 44%), after applying the two-fold criterion the number of under-expressed probesets with decreased intensity in neoplastic tissues was much larger than the number of probesets with increased intensity, 338 (76%) versus 108 (24%) respectively. The trend was observed in both non-neoplastic versus adenoma and non-neoplastic versus cancer comparisons. On the other hand, comparison of normal and colitis specimens showed approximately equal numbers of genes with higher (60) and lower (73) expression levels between phenotypes. Between adenoma and cancers, however, there were considerably more genes up-regulated (145) in cancer versus down regulated (43). A summary of differential expression change by phenotype is shown in Table 1 and a list of validated genes up- and down-regulated in cancers compared to adenomas are shown in Tables S1 and S2, respectively.

Probesets that revealed differential expression between neoplastic (adenomas and cancers) and non-neoplastic tissues (normal and colitis) were mapped to putative gene symbols using the most recent microarray annotation files. 108 probesets elevated in neoplastic tissues by at least two-fold were mapped to 97 gene symbols and 338 decreased probesets were mapped to 264 gene symbols (Table S3).

### Phenotype-specific genes

**Figure 2:**
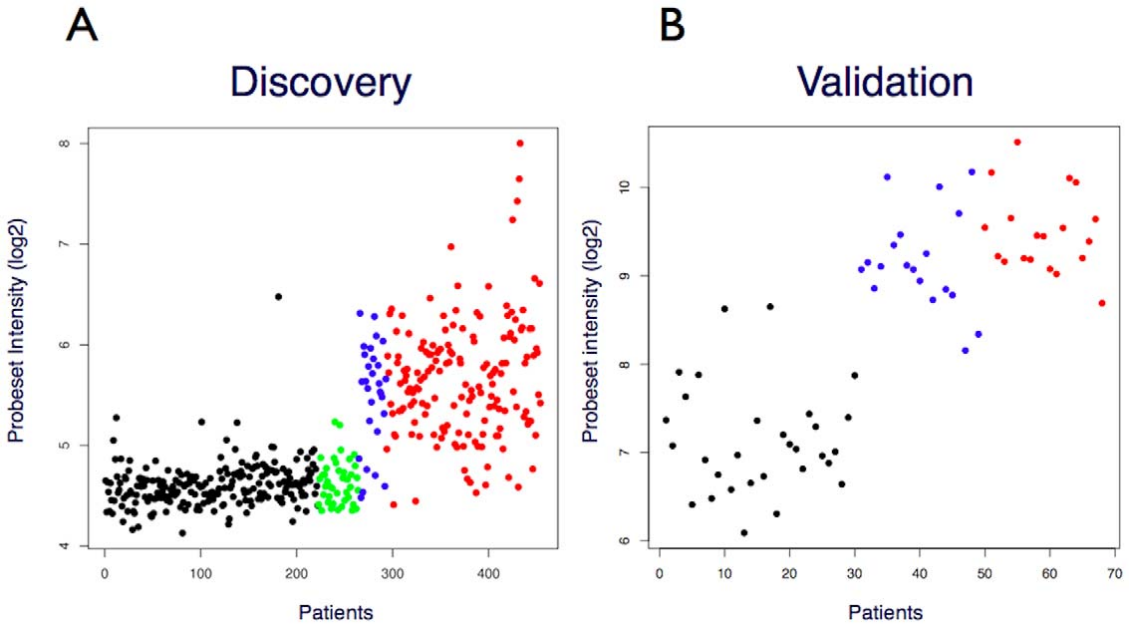
NFE2L3 – prototypical ‘switched ON’ gene in colorectal neoplastic tissue relative to non-neoplastic tissue specimens. (A) Discovery data set, 222 normal, black; 42 IBDs, green; 29 adenomas, blue; 161 adenocarcinomas, red. (B) Validation data set: 30 normal, black; 19 adenomas, blue; 19 adenocarcinomas, red. Y-axis: Normalized probeset intensity (log2).

The sets of differentially expressed genes were further analysed using a new analysis method developed by us to predict transcripts that may be expressed in one phenotype (e.g. neoplasia) but not in another (e.g. healthy controls). By applying this methodology, 23 probeset targets were identified as putative candidates for neoplastic-specific gene expression, i.e. hypothetically switched-on in neoplastic tissues but switched-off in non-neoplastic controls. In addition, 35 genes were identified as candidates for expression in non-neoplastic tissues only, i.e. switched-on in non-neoplastic tissues but switched-off in neoplastic tissues. An example of a probeset exhibiting a prototypical neoplastic-specific response pattern is shown in Figure 2A, and the complete list of probesets corresponding to hypothetically switched-on and switched-off genes is shown in Tables S4 and S5, respectively.

### Custom “Adenoma Biomarker” gene chip

The PCA plot of the discovery data (Fig 1A) shows a strong neoplasia vs. non-neoplasia segregation involving the first two principal component axes, with the adenomas and cancer grouped together. For the validation data (Fig 1B), the first principal component confirms that the largest source of variance across these probesets is the presence or absence of neoplasia. The second principal component, however, shows that the adenomas are grouped separately from the cancer specimens.

### Validation of candidate biomarkers for colorectal neoplasia

Of the 108 probesets whose targets were hypothesized to be over-expressed by at least two fold in neoplastic discovery tissues, 103 probesets (95%) were elevated in the neoplastic validation tissues (*P*≤0.05, MHT), and of these 92 (85%) were elevated by at least 2-fold. Similarly, 297 of the 338 (87%) probeset targets hypothesized to be under-expressed in neoplastic tissues were also under-expressed in the validation experiments (*P*≤0.05, MHT); in neoplastic specimens, 247 (73%) of these genes exhibited half or less of the expression seen in normal control tissues. Validation results by phenotype contrast are shown in Table 2. A list of validated up- and down-regulated probesets is shown in Tables S6 and S7, respectively.

**Table 2:** Summary of microarray discovery and validation studies.

		Discovery		Validation	
		Sig.Mean Diff & Diff FC > = 2		Sig Mean Diff	
Class A	Class B	Up	Down	Up	Down
Normal	Adenoma & Cancer	108	338	103	297
Normal	Adenoma	106	383	103	284
Normal	Cancer	158	371	134	306
Adenoma	Cancer	145	43	58	25

Review of probeset numbers for hypothesis discovery and hypothesis validation data sets. Note that an ‘up’ probeset means a probeset response differentially higher in the Class B phenotype relative to the Class A phenotype.

### Quantitative PCR assay for measuring RNA biomarker levels in tissue or plasma

**Figure 3:**
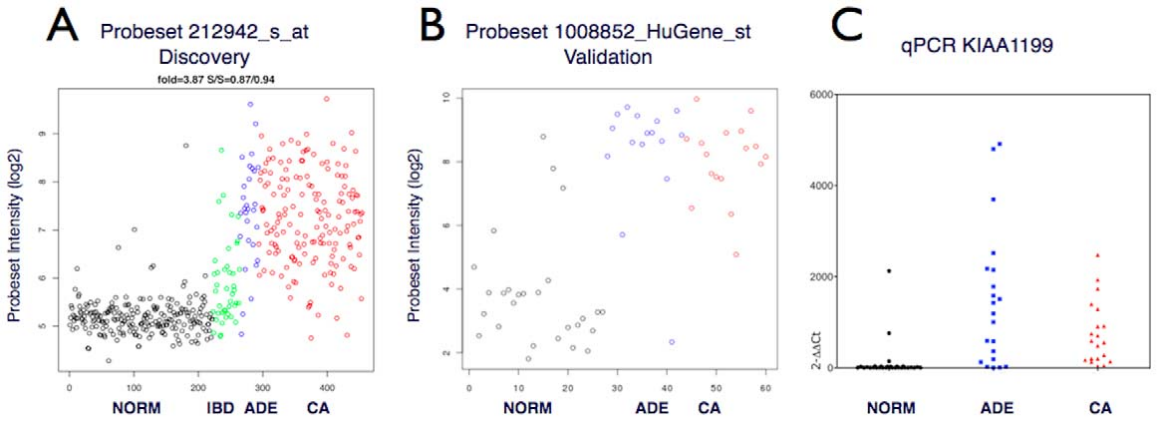
*KIAA1199* expression in colon tissue specimens. (**A**) *KIAA1199* expression measured via probeset 212942_s_at in the discovery dataset of 454 colorectal tissue specimens (x-axis indexed by phenotype); Norm: 222 normal specimens, black; IBD: 42 ‘colitis’ specimens, green; ADE: 29 adenomas, blue; CA: 161 cancer specimens, red. Y-axis: normalized probeset intensity (log2). (**B**) *KIAA1199* expression measured via probeset 1008852-HuGene_st in the validation dataset in 68 colorectal tissue specimens. X-axis; Norm: 30 normal colon tissue specimens, black; ADE: 19 adenomas, blue; CA: 19 colorectal cancer specimens, red. Y-axis: normalized probeset intensity (log2). (**C**) A quantitative real-time SYBR-green *KIAA1199* PCR assay applied to RNA extracts used for the validation microarray data: 30 normal, black; 21 adenomas, blue; 20 colorectal cancer, red specimens. Data are mean values of duplicates, normalized against HPRT1 and depicted as delta-delta-Ct values. Note that three additional neoplastic specimens were available for the PCR experiments which were not tested by custom microarray.

One of the most differentially up-regulated probesets in both the discovery (Fig 3A) and validation (Fig 3B) detected transcripts from *KIAA1199*, a gene of unknown function. In the validation data set, probeset 1008852-HuGene_st (*KIAA1199*) was expressed more than 25-fold higher in colorectal neoplasia relative to non-neoplastic controls (Table S6). These results confirmed earlier reports, which found that *KIAA1199* mRNA may be a candidate biomarker for colorectal adenoma [14]. Based on our repeated observation of differential expression in neoplastic tissues, the prior evidence of up-regulated expression in both adenomas and cancers and the interesting fact that *KIAA1199* has not been previously characterized in terms of structure or function, we chose *KIAA1199* to test the idea that tissue expression patterns can be reflected in blood. To further explore the biomarker potential of *KIAA1199* we designed a real-time PCR assay for detection of RNA transcripts derived from this locus.

**Figure 4:**
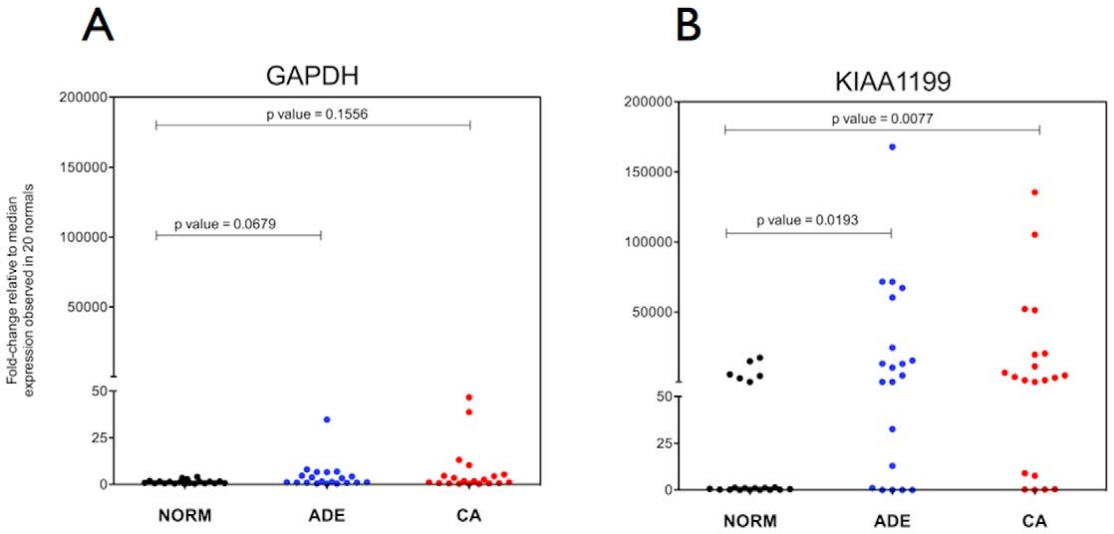
Measurement of RNA levels in plasma specimens. (**A**) *GAPDH* and (**B**) *KIAA1199* RNA levels in plasma from 20 healthy subjects (black) and from 20 patients with colon adenomas (blue) and 20 CRC patients (red). Data are mean Ct values (triplicates) normalized for extraction yield differences and depicted as fold-change differences relative to the median expression measured in the 20 normal subjects. P values were calculated using two-tailed Mann-Whitney t-test.

First, a SYBR-green based real-time PCR for *KIAA1199* was used to confirm the tissue validation microarray data; the results were in good agreement with the microarray data for this gene (Fig 3C). Next, *KIAA1199* (and *GAPDH* control) transcript levels were measured in RNA extracted from the plasma fraction of 40 patients with colorectal neoplasia (adenoma or adenocarcinoma) and 20 healthy controls (all categories having been confirmed by clinical pathological findings) using commercially available TaqMan qPCR assays. *GAPDH* RNA transcripts were detectable in all 60 plasma samples tested (Fig 4A) and moderately higher *GAPDH* RNA levels were observed in plasma specimens from patients diagnosed with colorectal adenomas or cancer compared to healthy donors (not significant, p values >0.05). Higher concentrations of *KIAA1199* RNA transcripts were detected in plasma from patients with colorectal neoplasia than in plasma from healthy controls (Fig 4B). *KIAA1199* RNA was detected in plasma from 31 out of the 40 (77.5%) patients with colorectal neoplasia and in 6 out of the 20 (30%) neoplasia-free patients.

## Discussion

This study confirms that colorectal mRNA transcripts are differentially expressed in both adenoma and cancer tissues relative to controls, with some transcripts being up-regulated in neoplasia while others are down-regulated. We confirmed that 103 of 108 (95%) probesets discovered to be up-regulated in neoplasia were likewise differentially expressed during validation testing. 87% (297/338) of the down-regulated probesets were also confirmed during validation testing. Genome-wide covariance patterns showed that the presence or absence of neoplasia correlated with the largest source of variance across all probesests and all arrays in the expression data (Fig 1A). Approximately 25% of the 44,928 discovery probeset targets were differentially expressed between neoplastic tissue and non-neoplastic controls. All other phenotype contrasts resulted in fewer probesets showing a differential response. These results demonstrate that neoplastic status has a larger influence on gene expression than, for example, colitis or even the difference between pre-invasive adenoma tissue and malignant cancer.

Our results agree with a commonly observed trend in colorectal cancer gene expression research that shows a higher number of genes are down-regulated in adenoma and cancer tissues compared to non-neoplastic controls [15]. This expression pattern may reflect increased levels of hypermethylation associated with oncogenesis [16].

On the other hand, this study reveals that more genes appear to be up-regulated in the transition from adenoma to adenocarcinoma (Table S1). This observation could reflect underlying increased histological complexity of cancer compared to adenoma tissue or, more interestingly, may demonstrate a relationship between increased numbers of expressed genes and the progression to an invasive phenotype and metastasis. The largest group (14%) of genes up-regulated in cancer compared to adenomas are from the collagen family but the list also includes four different species of matrix metaloproteinases suggesting increased activity of genes with invasion potential (Table S1).

Furthermore, this study involved the design of a custom microarray that contained a set of genes showing that adenomas can be separated from cancer specimens based on gene expression patterns (Fig 1B). These results support the concept that not only is the neoplastic gene expression signature conserved between the discovery and validation data but also the adenoma vs. cancer expression signature is likewise preserved. We are not aware of any previous gene expression study that has demonstrated the capacity to distinguish between non-neoplasia, pre-invasive neoplasia and invasive phenotypes. This selection of genes opens the way for the identification of biomarkers of use in the sensitive and specific detection of adenomas.

The second aim of this study was to explore whether selected candidate biomarkers discovered in tissue were detectable and differentially expressed in the plasma of patients with adenomas or colorectal cancer compared to non-neoplastic control plasma. This step is crucial for translation of tissue findings into clinically useful endpoints. Otherwise, marker discovery must start in diagnostic clinical specimens (e.g. blood) which pose greater challenges than starting with relatively RNA-rich fresh frozen tissue. This report describes a proof-of-concept plasma-based qPCR assay that measures mRNA transcripts of *KIAA1199*, a gene of unknown function that we confirm to be differentially expressed in both tissue and plasma of cancer and adenoma patients.

### Biomarkers for colorectal neoplasia

There is a large and growing literature of colorectal gene expression-related experiments [17], [18]. The study presented here extends and improves upon that body of work. A comparatively large meta-analysis by Chan et al. of 25 gene expression discovery studies related to colorectal cancer identified five genes to be up-regulated in seven or more independent analyses, including *TGFBI*, *IFITM1*, *MYC*, *SPARC*, *GDF15* [17]. All five of these genes were confirmed to be up-regulated in our study.

Few studies address expression differences between colorectal adenomas and normal colorectal tissue. Galamb et al. used microarrays to identify a set of three genes (*KIAA1199*, *FOXQ1*, and *CA7*) that were differentially expressed in adenomas relative to normal controls, as well as a set of five genes (*VWF*, *IL8*, *CHI3L1*, *S100A8*, and *GREM1*) which could discriminate cancer tissues from normal controls [19]. Of these genes, our study found that *KIAA1199*, *FOXQ1* and *IL8* were differentially expressed in adenomas (and in cancers) relative to normal controls.

### KIAA1199

The present study confirms earlier reports that *KIAA1199* exhibits an elevated level of mRNA expression in precancerous adenomas, an up-regulation that persists in cancerous tissue [14]. This gene was also one of the top markers identified by Marra's laboratory as a previously unknown target of Wnt-induced expression and a possible novel biomarker for colorectal neoplasia. Sabates-Bellver et al. demonstrated that *KIAA1199* expression in normal mucosa was confined to cells in the lower portion of intestinal crypts, whereas elevated *KIAA1199* expression was observed in all of the adenomas that they studied.

The role of *KIAA1199* is not known, but the evidence of Wnt-inducibility suggests this gene may be part of the downstream cascade of Tcf/LEF transcriptionally activated genes which are commonly perturbed in gastrointestinal neoplasia [14]. Gastric adenocarcinomas expressing high levels of *KIAA1199* are correlated with worse five-year survival outcomes relative to those patients with low *KIAA1199* expression [20]. Colon cancer cells treated with selective cyclooxygenase-2 inhibitors show lowered *KIAA1199* expression [21], while high levels of *KIAA1199* mRNA are positively correlated with cell mortality in human fibroblasts [22].

### A proof-of-concept blood test for adenomas

Despite the rapidly growing database of putative cancer biomarkers, few promising candidates during initial discovery research survive subsequent validation testing with independent tissues. An even smaller fraction of candidates continue to show promise when those genes are selected for assay development and clinical testing. We have identified hundreds of biomarkers for colorectal neoplasia that have survived validation in independent clinical specimens. For one compelling neoplastic tissue biomarker, *KIAA1199*, we have also tested a single-gene qPCR assay that shows promise in discriminating between plasma samples from with patients with colorectal neoplasia and from healthy individuals. This gene, which was one of many “validated” genes, was chosen for further study based on the biomarker performance reported here, previous reports that showed *KIAA1199* to be elevated in colorectal neoplasia and because the biological function of this gene is unknown. Plasma *KIAA1199* levels were elevated in patients who had colonoscopy-confirmed adenomas or cancers relative to control plasma from neoplasia-free individuals, although the apparent sensitivity of this single marker was higher for cancer than for adenomas.

As a biomarker with the potential for the diagnosis in non-invasive patient samples, *KIAA1199* should now be considered for incorporation into mRNA (and possibly protein) assays and investigated in larger clinical and screening studies. Of particular interest will be the relationship of *KIAA1199* expression to the various genetic pathways of colorectal oncogenesis, such as the *Wnt* signalling pathway, which are commonly perturbed in colorectal cancers and adenomas.

Colorectal neoplasia exhibits characteristic patterns of gene expression. *KIAA1199* is differentially expressed in neoplastic tissues and *KIAA1199* transcripts are more abundant in the plasma of patients with either cancer or adenoma compared to controls. *KIAA1199* and other validated biomarkers described here warrant further evaluation as blood-based screening tests for colorectal neoplasia. A key challenge for this further evaluation will be to also address a test's relative sensitivities for adenomas and cancers given the much higher prevalence of benign neoplastic colorectal adenomas compared to malignant neoplastic colorectal carcinoma.

## Materials and Methods

All microarray data used in this study was documented in accordance with the MIAME standards for microarray experiments.

### Discovery data

Colorectal tissue specimens used for biomarker discovery were collected by Genelogic Inc (Gaithersburg, MD, USA) from patients who gave written consent according to ethics standards set by an independent review board composed of scientists and bioethicists who were not employees of Gene Logic. Each institution that supplied tissue samples to GeneLogic obtained informed consent from each donor or, if applicable, their authorized representative, and met the requirements of the relevant Institutional Review Board and all applicable laws, Gene expression profiling data measured in 548 colorectal tissue specimens using Affymetrix HGU133A & HGU133B gene chips (44,928 probesets combined) (Affymetrix, Santa Clara, CA, USA) and accompanying clinical data was purchased from GeneLogic Inc. Experimental and clinical descriptors were provided for all chip data files in addition to digitally archived microscopy images of histological preparations. Prior to carrying out discovery research using these data, rigorous quality control testing was applied to these data. A total of 454 microarrays met quality control requirements and were judged suitable for this discovery research. Further details of the quality control procedures applied to these data re provided in Supporting Information S1 Quality Control Analysis. Description of the tissue phenotypes for these discovery data is shown in Table 3 with cancer phenotype breakdown in Table 4.

**Table 3:** Phenotypic breakdown of clinical specimens used in this study.

Colon tissue specimens used in the ‘Discovery’ data					
		Normal	Colitis/IBD^1^	Adenoma	Cancer
Gender	Female	102 (46%)	17	16	93
	Male	120 (54%)	25	13	68
Anatomy	Proximal	70 (32%)	13	13	58
	Distal	95 (43%)	12	5	90
	Unknown	57 (26%)	17	11	13
Age	Under 50	48 (22%)	28 (67%)	6 (21%)	29 (18%)
	50–79	144 (65%)	14 (33%)	19 (66%)	109 (68%)
	Over 80	30 (14%)	1 (2%)	3 (10%	23 (14%)

Colon tissue specimens used in the ‘Validation’ data			
		Normal	Neoplasia^2^
Gender	Female	16 (53%)	20 (53%
	Male	14 (47%)	18 (47%)
Anatomy	Proximal	14 (47%)	18 (47%)
	Distal	16 (53%)	20 (53%
Age	Under 50	1 (3%)	3 (8%)
	50–79	25 (83%)	28 (74%)
	Over 80	4 (13%)	7 (18%)

Plasma specimens used for measuring *KIAA1199 RNA* levels in blood			
		Normal	Neoplasia^3^
Gender	Female	10 (50%)	28 (70%)
	Male	10 (50%)	12 (30%)
Age	Under 50	8 (40%)	8 (20%)
	50–79	12 (60%)	31 (78%)
	Over 80	0 (0%)	1 (3%)

1 42 Colitis/IBD: 2 Colitis; 13 Crohn's disease; 5 Diverticulitis of colon; 2 Proctitis; 20 Ulcerative colitis.

2 19 adenomas: 1 tubular adenomas, 8 tubulovillous adenomas, 2 villous adenomas, 2 familial adenomatous polyps, 6 unknown; 19 adenocarcinomas (17 Dukes' A and 2 Dukes' B).

3 20 adenomas: 11 tubular adenomas, 5 villous adenomas, 4 unknown; 20 adenocarcinomas (1 Dukes' A, 6 Dukes' B, 4 Dukes' C, 1 Dukes' D, 8 unknown). See Table 4 for a further breakdown of the cancer staging into T scores (a component of the TNM score).

**Table 4:** A description of tumor specimens by stage for discovery tissues.

TX stage	T1	T2	T3	T4	TIS	TX
Specimens	3	24	94	18	1	21

T stage detail (from TNM staging) for the specimens used for discovery by Affymetrix gene expression analysis. TX No description of the tumor's extent is possible because of incomplete information. Tis The cancer is in the earliest stage (in situ). It involves only the mucosa. It has not grown beyond the muscularis mucosa (inner muscle layer).T1 The cancer has grown through the muscularis mucosa and extends into the submucosa. T2 The cancer has grown through the submucosa and extends into the muscularis propria (thick outer muscle layer). T3 The cancer has grown through the muscularis propria and into the outermost layers of the colon or rectum but not through them. It has not reached any nearby organs or tissues. T4 The cancer has grown through the serosa (also known as the visceral peritoneum), the outermost lining of the intestines and may have grown through the wall of the colon or rectum to attach or invade nearby tissues.

### Phenotype-specific expression patterns

In addition to standard differential expression analysis, we introduced an analytical technique designed to filter differentially expressed probeset candidates for transcripts that we hypothesized were qualitatively “turned-on” in one phenotype class and qualitatively “turned-off” in a comparator phenotype. For this method, identification of “off” genes was based on the relatively simple assumption that most genes in a given tissue were *not* constitutively expressed above a nominal relatively low background level. Consequently, a microarray designed to hybridize to the full human transcriptome should therefore not exhibit transcript-specific binding for most probesets in a given experiment. Conversely, the fluorescent intensity of probesets that hybridized to the balance of non-expressed transcripts should reflect “non-specific” probeset-transcript hybridization. The assumption that a large fraction of the probesets for any given experiment were not transcript-specific signals provided means to estimate a theoretical on/off threshold for genes in full-genome experiments such as used here for discovery. The mean expression level for all 44,928 probesets in the 454 discovery microarrays were ranked and the probeset value corresponding to the 30^th^ percentile value across the data was chosen as the threshold for transcriptional silence. This threshold represents a conservative upper-bound estimate of non-specific or background expression.

### Validation Data: Tissue specimens

For all validation (i.e. hypothesis testing) experiments, independently collected fresh frozen tissue specimens were obtained from a tertiary referral hospital tissue bank (Flinders Medical Centre, Adelaide, SA Australia). A description of cases used for validation testing is shown in Table 3. This study was approved by the Research and Ethics Committee of the Repatriation General Hospital and the Ethics Committee of Flinders Medical Centre. Written informed patient consent was received for each tissue studied. Surgical specimens were collected and processed as previously described [23].

### Validation Data: Custom microarray design

To test the many hypothetical gene biomarkers identified by discovery analyses, a custom-designed microarray, the “Adenoma Biomarker Gene Chip”, was fabricated (Affymetrix). The Adenoma Biomarker Gene Chip array included all HGU133-A/B probesets identified during discovery as well as exon-level probesets that were not available at the time of the original discovery exercise. Each HGU133 discovery probeset on the Adenoma Biomarker Gene Chip was annotated to one or more human gene symbols based on NCBI annotation tools (NCBI36/hg18) and these gene symbols were then reverse mapped back to exon-level probesets designed by Affymetrix for the HuGene ST 1.0 GeneChip. The custom microarray included “perfect-match” probesets only.

### Validation Data: RNA Extraction

A phenotypic breakdown of tissues used for validation testing is shown in Table 3. RNA was extracted from frozen tissue samples using Trizol (Invitrogen, San Diego USA) as recommended by manufacturer. Briefly, frozen tissues were homogenized in 300 µL of Trizol reagent using a modified Dremel drill and sterile disposable pestles. 200 µL of Trizol reagent was added to the homogenate and samples were incubated at room temperature (RT: 25C) for 10 minutes. 100 µL of (% v/v) chloroform was then added, samples were shaken for 15 seconds, and incubated at RT for 3 minutes. The aqueous phase containing total RNA was obtained by centrifugation at 12,000 x g for 15 min, 4°C. RNA was then precipitated by incubating samples at RT for 10 min with 250 µL isopropanol. Purified RNA precipitate was collected by centrifugation at 12,000 x g for 10 minutes, 4°C and supernatants were discarded.

### Adenoma Biomarker Gene Chip processing

The custom microarrays were processed using standard Affymetrix protocols developed for the HuGene ST 1.0 array as previously described [24]. The resulting expression data files are available in the Gene Expression Omnibus database (accession number GSE24713).

### Measurement of *KIAA1199* RNA expression in colon tissue specimens by quantitative PCR

1 µg of RNA extracted from validation tissues as described above was converted to cDNA in a 20 µl reaction using a High Capacity cDNA Reverse Transcription Kit with random primers (Applied Biosystems, Foster City, CA US). The reverse transcription reaction was diluted two-fold with RNase-free water.

Specific intron-spanning primers were designed for *KIAA1199* (forward primer [FWD]: CTG AAG CAT ATG GGA CAG CA and reverse primer [REV]: AGC AGT GGC CCA AAG AGT TA) and HPRT1 (FWD: TGA CAC TGG CAA AAC AAT GCA and REV: GGT CCT TTT CAC CAG CAA GCT). PCR reactions were carried out in duplicate in a final volume of 10 µl containing 5 µl 2X PCR Mastermix (Promega, Madison, WI US), 0.25 µ l of 1∶3000 diluted SYBR Green Nucleic Acid Stain (Invitrogen, San Diego, CA US), 0.6 µl of both forward and reverse primers (final conc. 300 nM), 2.55 µl of nuclease-free water and 1 µl of cDNA. Real-time PCR (qPCR) was performed on a Light Cycler LC480 (Roche, Basel, Switzerland) using the following cycling conditions: 95°C for 5 min, 40 cycles of 95°C for 15 sec and 60° for 1 min. Specificity of qPCR reactions was assessed by visual inspection of melting curves and by agarose-EtBr gel electrophoretic visualization of resulting qPCR products. qPCR assay specificity was ascertained by isolation and subsequent sequencing of qPCR products according to standard laboratory procedures. Quantification of *KIAA1199* expression was carried out using the comparative threshold method (2^–ΔΔCt^ value) using HPRT1 as an endogenous reference and a normal tissue sample as the calibrator [25].

### Plasma collection

Sixty plasma specimens were purchased through Proteogenex (Culver City, CA USA) from patients who gave written informed consent. Patient blood specimens were classified as normal (n = 20), adenoma (n = 20) or cancer (n = 20) patients based on colonoscopy results verified (where appropriate) by histopathology. Phenotype characteristics of the patients are given in Table 3. Blood was collected in K_3_EDTA vacutainer tubes and processed within 4 hours of blood draw. Plasma was generated by two consecutive 1,500 x g centrifugation spins for 10 min at 4°C. Resulting plasma was stored as 1 mL aliquots at −80°C until further use.

### Plasma RNA extraction

RNA was extracted from 2 mL plasma spiked with 2.5 µl of Armored RNA (armRNA) Enterovirus (Asuragen Diagnostics, Texas, US) using the QIAamp circulating nucleic acid kit (Qiagen, Hilden, Germany) as per manufacturer's instructions. RNA was eluted in 115 µl of AVE buffer and stored at −80°C until further use.

### Quantitative PCR analysis of RNA extracted from plasma specimens

30 µl of RNA extracted from 2 mL plasma was converted to a total of 60 µl of cDNA using the SuperScript VILO cDNA synthesis kit (Invitrogen, San Diego, US) as recommended by manufacturer. qPCR was performed using 2.5 µl (*KIAA1199* and *GAPDH* qPCR assays) or 0.25 µl cDNA (armRNA qPCR assay) in a final volume of 25 µl containing the EXPRESS qPCR Supermix Universal reagent (Invitrogen) and commercially available TaqMan assays for *KIAA1199* (Hs01552116_m1, Applied Biosystems, Foster City, CA US), GAPDH (Hs99999905_m1, Applied Biosystems) or primer/probe sequences for armRNA as previously described [26]. Reactions were run as triplicates. qPCR was performed on a Light Cycler LC480 (Roche) using the following cycle conditions: 50°C for 2 min and 95°C for 5 min, followed by 60 cycles of [95°C for 10 sec, 60°C for 50 sec, 72°C for 1 sec] then cooling to 40°C for 10 sec. Cycle threshold (Ct) values were calculated using absolute quantification / 2^nd^ derivative maximum. Patient mean Ct values (Ct_patient_) were normalised using mean Ct values obtained for the spiked in armRNA (Ct_armRNA patient_) and scaled relative to mean armRNA ct values obtained for the complete panel of plasma RNA samples analysed (Ct_armRNA complete panel_).

### Statistical methods

The R statistics environment was used for statistical analyses and open source libraries from BioConductor (Bioconductor, www.bioconductor.org) were used for analysing microarray data [27]–[29]. Affymetrix GCOS software was used to digitize arrays and raw CELDATA files were background corrected and normalized using the Robust Multichip Array (RMA) algorithm [29]. Probesets on the discovery and validation oligonucleotide microarrays were annotated to most likely gene symbol using the *hgu133plus2* library version 2.2.0 from BioConductor, assembled using Entrez Gene data downloaded on April 18, 2008.

To assess differential expression of probesets between phenotypes, Student's *t-*test for equal means between two samples as implemented in the *limma* library of R was used [30]. Multiple hypothesis test correction was applied using either Bonferonni (discovery) or Benjamini and Hochberg (validation) [31]–[33].

To evaluate the predictive accuracy of qPCR assays, logistic regression models were fitted to the cycle threshold data. A neoplasia classification (adenoma or cancer) was applied if the model predicted probability of the fitted regression value for that tissue was greater than or equal to 50%.

## Supporting Information

Table S1 Probesets identified to be at least two-fold up-regulated in colorectal cancer (n = 161) relative to adenoma (n = 29) tissue specimens.
(DOC)

Table S2 Probesets identified to be at least two-fold down-regulated in colorectal cancer (n = 161) relative to adenoma (n = 29) tissue specimens.
(DOC)

Table S3 Genes identified to be at least two-fold differentially expressed in colorectal neoplastic (29 adenomas + 161 cancers) relative to non-neoplastic (222 normals + 42 IBDs) tissue specimens.
(DOC)

Table S4 Discovery Probesets hypothesized to be switched-on in colorectal neoplastic tissues relative to non-neoplastic tissues.
(DOC)

Table S5 Discovery Probesets hypothesized to be switched-off in colorectal neoplastic tissues relative to non-neoplastic tissues.
(DOC)

Table S6 Confidence intervals of sensitivity and specificity for each validated up-regulated probeset in colorectal neoplasia (19 adenomas + 19 cancers) relative to 30 normal colon tissue specimens (validation data). Note that sensitivity and specificity calculations are estimated based from the mid-point of ROC curves (approximate inflection point) and are included for comparison purposes only.
(DOC)

Table S7 Confidence intervals of sensitivity and specificity for each validated down-regulated probeset target in colorectal neoplasia (19 adenomas + 19 cancers) relative to 30 normal colon tissue specimens. Note that sensitivity and specificity calculations are estimated based from the mid-point of ROC curves (approximate inflection point) and are included for comparison purposes only.
(DOC)

Supporting Information S1 **Quality Control Analysis.** This document details the quality control methods applied to the discovery data to assess Gene Chip quality.
(PDF)
