# Supplementary material for: Discovery and Validation of Molecular Biomarkers for Colorectal Adenomas and Cancer with Application to Blood Testing
Source: PLoS One. 2012 Jan 19;7(1):e29059. doi: 10.1371/journal.pone.0029059 (PMC3261845; doi:10.1371/journal.pone.0029059)
Supplement: Table S1 — Probesets identified to be at least two-fold up-regulated in colorectal cancer (n = 161) relative to adenoma (n = 29) tissue specimens. (DOC) [file pone.0029059.s001.doc]

**SUPPLEMENTARY TABLE S1.** Probesets identified to be at least two-fold up-regulated in colorectal cancer (n=161) relative to adenoma (n=29) tissue specimens.

SUPPLEMENTAL TABLE S1

| ProbeSetID | Symbol | | | Fold-∆(log2) | | t statistic | P value  (Bonf. Corr) | | | Likelihood |
| --- | --- | --- | --- | --- | --- | --- | --- | --- | --- | --- |
| 202404_s_at | | COL1A2 | 3.26 | | 14.42 | | | 9.57E-28 | 62.03 | |
| 202310_s_at | | COL1A1 | 3.06 | | 13.87 | | | 4.40E-26 | 58.37 | |
| 200665_s_at | | SPARC | 2.26 | | 12.86 | | | 4.84E-23 | 51.65 | |
| 215076_s_at | | COL3A1 | 2.4 | | 12.41 | | | 1.10E-21 | 48.65 | |
| 202403_s_at | | COL1A2 | 2.38 | | 12.41 | | | 1.11E-21 | 48.64 | |
| 210495_x_at | | FN1 | 2.82 | | 12.39 | | | 1.32E-21 | 48.47 | |
| 212464_s_at | | FN1 | 2.95 | | 12.35 | | | 1.73E-21 | 48.21 | |
| 211719_x_at | | FN1 | 2.97 | | 12.33 | | | 2.02E-21 | 48.07 | |
| 216442_x_at | | FN1 | 2.77 | | 12.09 | | | 1.03E-20 | 46.5 | |
| 201852_x_at | | COL3A1 | 2.4 | | 11.86 | | | 5.12E-20 | 44.96 | |
| 211980_at | | COL4A1 | 1.54 | | 11.55 | | | 4.29E-19 | 42.91 | |
| 211161_s_at | | COL3A1 | 2.38 | | 11.04 | | | 1.41E-17 | 39.55 | |
| 225681_at | | CTHRC1 | 3.01 | | 10.98 | | | 2.07E-17 | 39.18 | |
| 201438_at | | COL6A3 | 1.98 | | 10.96 | | | 2.30E-17 | 39.08 | |
| 221729_at | | COL5A2 | 2.26 | | 10.67 | | | 1.65E-16 | 37.18 | |
| 212354_at | | SULF1 | 2.24 | | 10.58 | | | 3.11E-16 | 36.57 | |
| 210809_s_at | | POSTN | 2.84 | | 10.54 | | | 4.05E-16 | 36.32 | |
| 221731_x_at | | VCAN | 2.17 | | 10.36 | | | 1.33E-15 | 35.17 | |
| 211981_at | | COL4A1 | 1.54 | | 10.25 | | | 2.74E-15 | 34.48 | |
| 211964_at | | COL4A2 | 1.48 | | 10.22 | | | 3.34E-15 | 34.28 | |
| 218638_s_at | | SPON2 | 1.21 | | 10 | | | 1.45E-14 | 32.87 | |
| 202998_s_at | | LOXL2 | 1.38 | | 9.96 | | | 1.98E-14 | 32.57 | |
| 201744_s_at | | LUM | 2.22 | | 9.67 | | | 1.34E-13 | 30.73 | |
| 201162_at | | IGFBP7 | 1.34 | | 9.57 | | | 2.54E-13 | 30.11 | |
| 204620_s_at | | VCAN | 1.98 | | 9.5 | | | 3.91E-13 | 29.7 | |
| 227140_at | | -NA- | 2.33 | | 9.49 | | | 4.29E-13 | 29.61 | |
| 201105_at | | LGALS1 | 1.51 | | 9.4 | | | 7.51E-13 | 29.07 | |
| 211959_at | | IGFBP5 | 2.03 | | 9.28 | | | 1.63E-12 | 28.32 | |
| 208788_at | | ELOVL5 | 1.5 | | 9.23 | | | 2.36E-12 | 27.96 | |
| 212667_at | | SPARC | 1.81 | | 9.13 | | | 4.51E-12 | 27.34 | |
| 221011_s_at | | LBH | 1.16 | | 9.1 | | | 5.25E-12 | 27.19 | |
| 208782_at | | FSTL1 | 1.33 | | 9.07 | | | 6.54E-12 | 26.98 | |
| 213905_x_at | | BGN | 1.62 | | 9.03 | | | 8.24E-12 | 26.76 | |
| 212489_at | | COL5A1 | 1.6 | | 8.99 | | | 1.08E-11 | 26.5 | |
| 217764_s_at | | RAB31 | 1.56 | | 8.9 | | | 1.86E-11 | 25.98 | |
| 225664_at | | COL12A1 | 1.93 | | 8.9 | | | 1.86E-11 | 25.97 | |
| 218468_s_at | | GREM1 | 2.2 | | 8.81 | | | 3.34E-11 | 25.41 | |
| 221730_at | | COL5A2 | 1.92 | | 8.79 | | | 3.80E-11 | 25.29 | |
| 217762_s_at | | RAB31 | 1.55 | | 8.79 | | | 3.80E-11 | 25.29 | |
| 212488_at | | COL5A1 | 1.6 | | 8.79 | | | 3.96E-11 | 25.25 | |
| 212353_at | | SULF1 | 1.99 | | 8.77 | | | 4.41E-11 | 25.14 | |
| 202311_s_at | | COL1A1 | 2.19 | | 8.71 | | | 6.37E-11 | 24.79 | |
| 226311_at | | -NA- | 1.4 | | 8.7 | | | 6.82E-11 | 24.72 | |
| 210511_s_at | | INHBA | 1.7 | | 8.7 | | | 6.83E-11 | 24.72 | |
| 203477_at | | COL15A1 | 1.88 | | 8.59 | | | 1.38E-10 | 24.05 | |
| 208851_s_at | | THY1 | 1.02 | | 8.56 | | | 1.64E-10 | 23.88 | |
| 207173_x_at | | CDH11 | 1.9 | | 8.52 | | | 2.16E-10 | 23.61 | |
| 217763_s_at | | RAB31 | 1.54 | | 8.49 | | | 2.58E-10 | 23.44 | |
| 213869_x_at | | THY1 | 1.13 | | 8.35 | | | 5.92E-10 | 22.64 | |
| 218469_at | | GREM1 | 2.01 | | 8.23 | | | 1.29E-09 | 21.9 | |
| 212344_at | | SULF1 | 1.48 | | 8.17 | | | 1.81E-09 | 21.57 | |
| 202450_s_at | | CTSK | 1.2 | | 8.11 | | | 2.67E-09 | 21.19 | |
| 201069_at | | MMP2 | 1.45 | | 8.08 | | | 3.23E-09 | 21.01 | |
| 201185_at | | HTRA1 | 1.29 | | 8.02 | | | 4.73E-09 | 20.64 | |
| 211966_at | | COL4A2 | 1.3 | | 7.96 | | | 6.85E-09 | 20.29 | |
| 203083_at | | THBS2 | 1.89 | | 7.94 | | | 7.53E-09 | 20.19 | |
| 225799_at | | LOC541471 | 1.03 | | 7.93 | | | 8.19E-09 | 20.11 | |
| 226930_at | | FNDC1 | 1.77 | | 7.92 | | | 8.57E-09 | 20.07 | |
| 212077_at | | CALD1 | 1.66 | | 7.9 | | | 9.70E-09 | 19.95 | |
| 226237_at | | COL8A1 | 1.94 | | 7.84 | | | 1.34E-08 | 19.64 | |
| 201261_x_at | | BGN | 1.22 | | 7.83 | | | 1.44E-08 | 19.57 | |
| 200832_s_at | | SCD | 1.26 | | 7.8 | | | 1.74E-08 | 19.39 | |
| 231766_s_at | | COL12A1 | 1.6 | | 7.8 | | | 1.77E-08 | 19.37 | |
| 208850_s_at | | THY1 | 1.13 | | 7.79 | | | 1.85E-08 | 19.33 | |
| 209875_s_at | | SPP1 | 2.68 | | 7.77 | | | 2.06E-08 | 19.23 | |
| 224724_at | | SULF2 | 1.22 | | 7.72 | | | 2.85E-08 | 18.91 | |
| 201163_s_at | | IGFBP7 | 1.3 | | 7.72 | | | 2.92E-08 | 18.89 | |
| 224694_at | | ANTXR1 | 1.64 | | 7.54 | | | 8.47E-08 | 17.87 | |
| 231579_s_at | | TIMP2 | 1.37 | | 7.46 | | | 1.36E-07 | 17.41 | |
| 219087_at | | ASPN | 1.98 | | 7.42 | | | 1.71E-07 | 17.19 | |
| 213428_s_at | | COL6A1 | 1.21 | | 7.38 | | | 2.10E-07 | 17 | |
| 200600_at | | MSN | 1.14 | | 7.35 | | | 2.45E-07 | 16.85 | |
| 202878_s_at | | CD93 | 1 | | 7.31 | | | 3.23E-07 | 16.58 | |
| 203878_s_at | | MMP11 | 1.05 | | 7.3 | | | 3.42E-07 | 16.53 | |
| 205479_s_at | | PLAU | 1.04 | | 7.29 | | | 3.48E-07 | 16.51 | |
| 201426_s_at | | VIM | 1.28 | | 7.28 | | | 3.70E-07 | 16.45 | |
| 214247_s_at | | DKK3 | 1.18 | | 7.27 | | | 3.99E-07 | 16.38 | |
| 210095_s_at | | IGFBP3 | 1.14 | | 7.2 | | | 6.01E-07 | 15.98 | |
| 203325_s_at | | COL5A1 | 1.11 | | 7.18 | | | 6.63E-07 | 15.89 | |
| 209156_s_at | | COL6A2 | 1.68 | | 7.17 | | | 7.07E-07 | 15.83 | |
| 224560_at | | TIMP2 | 1.27 | | 7.15 | | | 8.22E-07 | 15.68 | |
| 209218_at | | SQLE | 1.15 | | 7.08 | | | 1.19E-06 | 15.32 | |
| 202766_s_at | | FBN1 | 1.34 | | 7.03 | | | 1.57E-06 | 15.06 | |
| 201141_at | | GPNMB | 1.63 | | 7.02 | | | 1.68E-06 | 15 | |
| 207191_s_at | | ISLR | 1.12 | | 6.98 | | | 2.16E-06 | 14.76 | |
| 202859_x_at | | IL8 | 2.04 | | 6.98 | | | 2.16E-06 | 14.76 | |
| 202237_at | | NNMT | 1.45 | | 6.97 | | | 2.26E-06 | 14.71 | |
| 209955_s_at | | FAP | 1.28 | | 6.95 | | | 2.55E-06 | 14.6 | |
| 211896_s_at | | DCN | 1.84 | | 6.94 | | | 2.62E-06 | 14.57 | |
| 213125_at | | OLFML2B | 1.1 | | 6.88 | | | 3.70E-06 | 14.24 | |
| 227566_at | | HNT | 1.11 | | 6.87 | | | 4.05E-06 | 14.15 | |
| 201147_s_at | | TIMP3 | 1.26 | | 6.84 | | | 4.61E-06 | 14.03 | |
| 201150_s_at | | TIMP3 | 1.33 | | 6.83 | | | 4.87E-06 | 13.98 | |
| 204475_at | | MMP1 | 2.33 | | 6.83 | | | 4.97E-06 | 13.96 | |
| 233555_s_at | | SULF2 | 1.04 | | 6.82 | | | 5.21E-06 | 13.91 | |
| 208747_s_at | | C1S | 1.18 | | 6.74 | | | 8.02E-06 | 13.5 | |
| 201792_at | | AEBP1 | 1.26 | | 6.7 | | | 1.03E-05 | 13.26 | |
| 204051_s_at | | SFRP4 | 1.73 | | 6.6 | | | 1.75E-05 | 12.75 | |
| 229802_at | | -NA- | 1.3 | | 6.54 | | | 2.42E-05 | 12.44 | |
| 209395_at | | CHI3L1 | 1.03 | | 6.54 | | | 2.45E-05 | 12.43 | |
| 201893_x_at | | DCN | 1.37 | | 6.51 | | | 2.90E-05 | 12.27 | |
| 209396_s_at | | CHI3L1 | 1.15 | | 6.42 | | | 4.81E-05 | 11.78 | |
| 215646_s_at | | VCAN | 1.43 | | 6.4 | | | 5.16E-05 | 11.72 | |
| 201616_s_at | | CALD1 | 1.36 | | 6.4 | | | 5.36E-05 | 11.68 | |
| 37892_at | | COL11A1 | 1.79 | | 6.39 | | | 5.55E-05 | 11.65 | |
| 202238_s_at | | NNMT | 1.23 | | 6.3 | | | 8.91E-05 | 11.19 | |
| 226694_at | | AKAP2 | 1.1 | | 6.28 | | | 9.99E-05 | 11.08 | |
| 201289_at | | CYR61 | 1.28 | | 6.26 | | | 0.0001 | 10.99 | |
| 231879_at | | COL12A1 | 1.34 | | 6.25 | | | 0.0001 | 10.93 | |
| 229218_at | | COL1A2 | 1.06 | | 6.24 | | | 0.0001 | 10.89 | |
| 209596_at | | MXRA5 | 1.19 | | 6.12 | | | 0.0002 | 10.26 | |
| 200974_at | | ACTA2 | 1.07 | | 6.09 | | | 0.0002 | 10.11 | |
| 226777_at | | -NA- | 1.11 | | 6.08 | | | 0.0002 | 10.09 | |
| 211571_s_at | | VCAN | 1.3 | | 6.08 | | | 0.0002 | 10.06 | |
| 225710_at | | GNB4 | 1.1 | | 5.95 | | | 0.0005 | 9.41 | |
| 209101_at | | CTGF | 1.16 | | 5.93 | | | 0.0006 | 9.32 | |
| 205547_s_at | | TAGLN | 1.59 | | 5.92 | | | 0.0006 | 9.26 | |
| 200986_at | | SERPING1 | 1.07 | | 5.87 | | | 0.0008 | 9.02 | |
| 202283_at | | SERPINF1 | 1.13 | | 5.86 | | | 0.0009 | 8.97 | |
| 204320_at | | COL11A1 | 1.02 | | 5.85 | | | 0.0009 | 8.94 | |
| 217430_x_at | | COL1A1 | 1.23 | | 5.81 | | | 0.0011 | 8.73 | |
| 218559_s_at | | MAFB | 1.04 | | 5.79 | | | 0.0013 | 8.63 | |
| 201667_at | | GJA1 | 1.26 | | 5.74 | | | 0.0016 | 8.4 | |
| 232458_at | | COL3A1 | 1.07 | | 5.67 | | | 0.0023 | 8.07 | |
| 223235_s_at | | SMOC2 | 1.29 | | 5.65 | | | 0.0025 | 7.99 | |
| 203570_at | | LOXL1 | 1 | | 5.64 | | | 0.0026 | 7.94 | |
| 211813_x_at | | DCN | 1.19 | | 5.62 | | | 0.0029 | 7.86 | |
| 204122_at | | TYROBP | 1.07 | | 5.58 | | | 0.0035 | 7.66 | |
| 223122_s_at | | SFRP2 | 1.99 | | 5.54 | | | 0.0043 | 7.48 | |
| 201645_at | | TNC | 1.15 | | 5.52 | | | 0.0049 | 7.36 | |
| 234994_at | | TMEM200A | 1.1 | | 5.5 | | | 0.0053 | 7.28 | |
| 202620_s_at | | PLOD2 | 1.06 | | 5.41 | | | 0.0083 | 6.86 | |
| 215049_x_at | | CD163 | 1.04 | | 5.39 | | | 0.0091 | 6.77 | |
| 201859_at | | SRGN | 1.17 | | 5.38 | | | 0.0094 | 6.74 | |
| 210764_s_at | | CYR61 | 1.06 | | 5.32 | | | 0.0131 | 6.43 | |
| 202917_s_at | | S100A8 | 1.78 | | 5.31 | | | 0.0134 | 6.41 | |
| 203645_s_at | | CD163 | 1.06 | | 5.27 | | | 0.0166 | 6.21 | |
| 201058_s_at | | MYL9 | 1.38 | | 5.26 | | | 0.0169 | 6.19 | |
| 227099_s_at | | LOC387763 | 1.03 | | 5.21 | | | 0.0215 | 5.96 | |
| 203382_s_at | | APOE | 1.14 | | 5.2 | | | 0.0232 | 5.89 | |
| 213524_s_at | | G0S2 | 1.15 | | 5.16 | | | 0.0271 | 5.74 | |
| 201842_s_at | | EFEMP1 | 1.14 | | 5.14 | | | 0.0306 | 5.63 | |
| 204006_s_at | | FCGR3B | 1.04 | | 5.1 | | | 0.0366 | 5.46 | |
| 205828_at | | MMP3 | 1.34 | | 5.1 | | | 0.0369 | 5.45 | |
| 202291_s_at | | MGP | 1.47 | | 5.08 | | | 0.0397 | 5.38 | |
