# Supplementary material for: Discovery and Validation of Molecular Biomarkers for Colorectal Adenomas and Cancer with Application to Blood Testing
Source: PLoS One. 2012 Jan 19;7(1):e29059. doi: 10.1371/journal.pone.0029059 (PMC3261845; doi:10.1371/journal.pone.0029059)
Supplement: Table S2 — Probesets identified to be at least two-fold down-regulated in colorectal cancer (n = 161) relative to adenoma (n = 29) tissue specimens. (DOC) [file pone.0029059.s002.doc]

**SUPPLEMENTARY TABLE S2.** Probesets identified to be at least two-fold down-regulated in colorectal cancer (n=161) relative to adenoma (n=29) tissue specimens.

**SUPPLEMENTAL TABLE S2**

| ProbeSetID | Symbol | Fold-∆(log2) | t statistic | P value  (Bonf. Corr) | Likelihood |
| --- | --- | --- | --- | --- | --- |
| 213106_at | ATP8A1 | −1.32 | −9.89 | 3.0825E-14 | 32.14 |
| 204811_s_at | CACNA2D2 | −1.09 | −9.71 | 1E-13 | 31.01 |
| 228232_s_at | VSIG2 | −1.60 | −9.65 | 1.4994E-13 | 30.62 |
| 235976_at | SLITRK6 | −1.37 | −8.45 | 3.2762E-10 | 23.21 |
| 232481_s_at | SLITRK6 | −1.67 | −8.03 | 4.3327E-09 | 20.73 |
| 208063_s_at | CAPN9 | −1.04 | −7.93 | 7.7912E-09 | 20.16 |
| 214234_s_at | CYP3A5P2 | −1.04 | −7.64 | 4.6538E-08 | 18.44 |
| 223970_at | RETNLB | −2.05 | −7.63 | 4.7545E-08 | 18.42 |
| 218211_s_at | MLPH | −1.16 | −7.46 | 1.3608E-07 | 17.41 |
| 232176_at | SLITRK6 | −1.33 | −7.45 | 1.3748E-07 | 17.4 |
| 204508_s_at | CA12 | −1.02 | −7.39 | 2.0392E-07 | 17.02 |
| 214235_at | CYP3A5P2 | −1.07 | −7.33 | 2.9079E-07 | 16.68 |
| 205765_at | CYP3A5 | −1.42 | −7.33 | 2.8178E-07 | 16.71 |
| 223969_s_at | RETNLB | −1.84 | −7.28 | 3.8401E-07 | 16.41 |
| 237521_x_at | -NA- | −1.07 | −7.27 | 3.9314E-07 | 16.39 |
| 205259_at | NR3C2 | −1.03 | −7.19 | 6.3752E-07 | 15.93 |
| 215125_s_at | UGT1A6 | −1.28 | −6.81 | 5.5498E-06 | 13.85 |
| 236894_at | L1TD1 | −1.30 | −6.70 | 9.9335E-06 | 13.29 |
| 203963_at | CA12 | −1.18 | −6.69 | 0.000010537 | 13.24 |
| 204897_at | PTGER4 | −1.18 | −6.57 | 0.000020624 | 12.59 |
| 221874_at | KIAA1324 | −1.03 | −6.48 | 0.000034133 | 12.11 |
| 204607_at | HMGCS2 | −1.95 | −6.39 | 0.00005621 | 11.63 |
| 219543_at | PBLD | −1.03 | −6.33 | 0.000078191 | 11.32 |
| 227719_at | -NA- | −1.19 | −6.30 | 0.000087743 | 11.21 |
| 200884_at | CKB | −1.37 | −6.23 | 0.0001 | 10.82 |
| 205927_s_at | CTSE | −1.40 | −6.13 | 0.0002 | 10.33 |
| 208937_s_at | ID1 | −1.44 | −6.02 | 0.0003 | 9.79 |
| 203240_at | FCGBP | −1.97 | −6.02 | 0.0004 | 9.75 |
| 210107_at | CLCA1 | −2.42 | −5.98 | 0.0004 | 9.6 |
| 215867_x_at | CA12 | −1.01 | −5.85 | 0.0009 | 8.94 |
| 219955_at | L1TD1 | −1.71 | −5.78 | 0.0013 | 8.59 |
| 217110_s_at | MUC4 | −1.08 | −5.67 | 0.0022 | 8.09 |
| 231832_at | GALNT4 | −1.02 | −5.66 | 0.0024 | 8.02 |
| 226248_s_at | KIAA1324 | −1.15 | −5.59 | 0.0034 | 7.71 |
| 229070_at | C6orf105 | −1.38 | −5.58 | 0.0036 | 7.64 |
| 226302_at | ATP8B1 | −1.14 | −5.45 | 0.007 | 7.02 |
| 227725_at | ST6GALNAC1 | −1.59 | −5.43 | 0.0077 | 6.94 |
| 242601_at | LOC253012 | −1.60 | −5.42 | 0.0079 | 6.91 |
| 214433_s_at | SELENBP1 | −1.22 | −5.41 | 0.0083 | 6.86 |
| 221841_s_at | KLF4 | −1.07 | −5.39 | 0.009 | 6.79 |
| 217109_at | MUC4 | −1.30 | −5.24 | 0.0191 | 6.07 |
| 204895_x_at | MUC4 | −1.11 | −5.24 | 0.0186 | 6.1 |
| 227676_at | FAM3D | −1.00 | −5.19 | 0.0242 | 5.85 |
