# Supplementary material for: Discovery and Validation of Molecular Biomarkers for Colorectal Adenomas and Cancer with Application to Blood Testing
Source: PLoS One. 2012 Jan 19;7(1):e29059. doi: 10.1371/journal.pone.0029059 (PMC3261845; doi:10.1371/journal.pone.0029059)
Supplement: Table S3 — Genes identified to be at least two-fold differentially expressed in colorectal neoplastic (29 adenomas + 161 cancers) relative to non-neoplastic (222 normals + 42 IBDs) tissue specimens. (DOC) [file pone.0029059.s003.doc]

**SUPPLEMENTARY TABLE S3.** Genes identified to be at least two-fold differentially expressed in colorectal neoplastic (29 adenomas + 161 cancers) relative to non-neoplastic (222 normals + 42 IBDs) tissue specimens.

SUPPLEMENTAL TABLE S3

| 97 up-regulated genes | | 264 down-regulated genes | | | |
| --- | --- | --- | --- | --- | --- |
| 7A5 | RNF43 | ABCA8 | DNASE1L3 | KRT20 | PRIMA1 |
| ANLN | RPL22L1 | ABCG2 | DPT | LGALS2 | PRKACB |
| ANXA3 | S100A11 | ABI3BP | EDG2 | LIFR | PTGER4 |
| ASCL2 | S100P | ACTG2 | EDIL3 | LMOD1 | PYY |
| AXIN2 | SCD | ADAMDEC1 | EDN3 | LOC253012 | RELL1 |
| AZGP1 | SERPINB5 | ADAMTS1 | EMP1 | LOC572558 | RGS2 |
| BGN | SERPINE2 | ADH1B | ENTPD5 | LOC646627 | RPL14 |
| C20orf199 | SFRP4 | ADH1C | EPB41L3 | LOC652128 | SCARA5 |
| CCL20 | SLC12A2 | AGPAT9 | ETHE1 | LOC96610 | SCNN1B |
| CCND1 | SLC39A10 | AHCYL2 | EXOC3 | LRRC19 | SDCBP2 |
| CDCA7 | SLC6A6 | AKR1B10 | FABP1 | LYVE1 | SDPR |
| CDH11 | SLC7A5 | ALDH1A1 | FABP4 | MAB21L2 | SELENBP1 |
| CDH3 | SLCO4A1 | ANGPTL1 | FAM107A | MALL | SEMA6D |
| CKS2 | SORD | ANK2 | FAM129A | MAMDC2 | SEPP1 |
| CLDN1 | SOX4 | ANK3 | FAM46C | MAOA | SFRP1 |
| COL11A1 | SOX9 | ANPEP | FAM55D | MATN2 | SGK1 |
| COL12A1 | SPP1 | AOC3 | FBLN1 | MEP1A | SI |
| COL1A1 | SQLE | AQP8 | FCGBP | METTL7A | SLC26A2 |
| COL1A2 | SULF1 | ARL14 | FERMT2 | MGC13057 | SLC26A3 |
| COL8A1 | TACSTD2 | ATP1A2 | FGL2 | MGC4172 | SLC4A4 |
| CSE1L | TCN1 | ATP8B1 | FHL1 | MGP | SMPDL3A |
| CST1 | TDGF1 | BCHE | FLNA | MIER3 | SMTN |
| CTHRC1 | TESC | BEST2 | FNBP1 | MMP28 | SORBS1 |
| CXCL1 | TGFBI | C10orf99 | FOSB | MPEG1 | SORBS2 |
| CXCL2 | THBS2 | C15orf48 | FOXF1 | MRGPRF | SPARCL1 |
| CXCL3 | TIMP1 | C17orf91 | FOXF2 | MS4A12 | SRPX |
| CXCL5 | TMEM97 | C1orf115 | FOXP2 | MSRB3 | SST |
| DPEP1 | TMEPAI | C2orf40 | FXYD6 | MT1E | ST6GALNAC1 |
| DUSP27 | TPX2 | C6orf105 | GCG | MT1F | STMN2 |
| ECT2 | TRIM29 | C7 | GCNT2 | MT1G | SULT1A1 |
| ENC1 | UBD | CA1 | GCNT3 | MT1H | SYNPO2 |
| FAM148A | UBE2C | CA12 | GPM6B | MT1M | TAGLN |
| FAP | WDR72 | CA2 | GPX3 | MT1X | TCF21 |
| FERMT1 |  | CA4 | GUCA1B | MUC12 | TMEM47 |
| FOXQ1 |  | CAV1 | GUCA2B | MUC4 | TNFRSF17 |
| GDF15 |  | CD177 | HBA1 | MUPCDH | TNS1 |
| GPR56 |  | CD36 | HBA2 | MYH11 | TNXB |
| hCG_1815491 |  | CDKN2B | HBB | MYL9 | TP53INP2 |
| HIG2 |  | CEACAM1 | HHLA2 | MYLK | TPM2 |
| IL8 |  | CEACAM7 | HIGD1A | NDE1 | TRPM6 |
| INHBA |  | CES2 | HLA-C | NR3C2 | TSC22D3 |
| JUB |  | CFD | HMGCS2 | NTN2L | TSPAN1 |
| *KIAA1199* |  | CFL2 | HPGD | OGN | TSPAN7 |
| KRT23 |  | CHGA | HSD11B2 | OSTbeta | UGDH |
| LCN2 |  | CHP2 | HSD17B2 | P2RY1 | UGP2 |
| LGR5 |  | CHRDL1 | HSPB6 | P2RY14 | UGT1A1 |
| LY6G6D |  | CITED2 | HSPB8 | PADI2 | UGT1A3 |
| MET |  | CLCA1 | IGHA1 | PAPSS2 | UGT1A6 |
| MMP1 |  | CLCA4 | IGHM | PCDH7 | UGT1A9 |
| MMP11 |  | CLDN23 | IGJ | PCK1 | UGT2A3 |
| MMP12 |  | CLDN8 | IGKV1D-13 | PDCD4 | UGT2B15 |
| MMP3 |  | CLEC3B | IGL@ | PDE9A | UGT2B17 |
| MMP7 |  | CLIC5 | IGLJ3 | PDK4 | VSIG2 |
| MSLN |  | CLU | IGLV1-44 | PGM5 | XDH |
| MTHFD1L |  | CMBL | IL1R2 | PIGR | ZCWPW2 |
| MYC |  | CNN1 | IL8 | PKIB | ZG16 |
| NEBL |  | CRYAB | ITLN1 | PLAC8 |  |
| NFE2L3 |  | CSRP1 | ITM2A | PLCE1 |  |
| PHLDA1 |  | CXCL12 | ITM2C | PLN |  |
| PSAT1 |  | DCN | IVD | POU2AF1 |  |
| PUS7 |  | DES | KCNMA1 | PPAP2A |  |
| REG1A |  | DHRS9 | KCNMB1 | PPID |  |
| REG1B |  | DMD | KCTD12 | PPP1R12B |  |
| REG3A |  | DMN | KLF4 | PPP1R14A |  |
