# Supplementary material for: Discovery and Validation of Molecular Biomarkers for Colorectal Adenomas and Cancer with Application to Blood Testing
Source: PLoS One. 2012 Jan 19;7(1):e29059. doi: 10.1371/journal.pone.0029059 (PMC3261845; doi:10.1371/journal.pone.0029059)
Supplement: Table S4 — Discovery Probesets hypothesized to be switched-on in colorectal neoplastic tissues relative to non-neoplastic tissues. (DOC) [file pone.0029059.s004.doc]

**SUPPLEMENTARY TABLE S4.** Discovery probesets hypothesized to be switched-on in colorectal neoplastic tissues relative to non-neoplastic tissues.

SUPPLEMENTAL TABLE S4

| Probeset | ID | Symbol |
| --- | --- | --- |
| 204702_s_at | NFE2L3 | nuclear factor (erythroid-derived 2)-like 3 |
| 227140_at | -NA- | -NA- |
| 225806_at | JUB | jub, ajuba homologue (Xenopus laevis) |
| 204259_at | MMP7 | matrix metalloproteinase 7 |
| 219787_s_at | ECT2 | epithelial cell transforming sequence 2 oncogene |
| 238021_s_at | hCG_1815491 | hCG1815491 |
| 213880_at | LGR5 | leu-rich rpt-containing G prot-coupled receptor 5 |
| 207850_at | CXCL3 | chemokine (C-X-C motif) ligand 3 |
| 37892_at | COL11A1 | collagen, type XI, alpha 1 |
| 222608_s_at | ANLN | anillin, actin binding protein |
| 202286_s_at | TACSTD2 | tumor-associated calcium signal transducer 2 |
| 241031_at | FAM148A | family with sequence similarity 148, member A |
| 206224_at | CST1 | cystatin |
| 209309_at | AZGP1 | alpha-2-glycoprotein, zinc-binding |
| 204475_at | MMP1 | matrix metalloproteinase 1 (interstitial collagenase) |
| 202311_s_at | COL1A1 | collagen, type I, alpha 1 |
| 227174_at | WDR72 | WD report domain 72 |
| 223062_s_at | PSAT1 | phosphoserine aminotransferase 1 |
| 226237_at | COL8A1 | collagen, type VIII, alpha 1 |
| 211506_s_at | IL8 | interleukin 8 |
| 232252_at | DUSP27 | dual specificity phosphatase 27 (putative) |
| 204885_s_at | MSLN | mesothelin |
| 214974_x_at | CXCL5 | chemokine (C-X-C motif) ligand 5 |
