# Supplementary material for: Discovery and Validation of Molecular Biomarkers for Colorectal Adenomas and Cancer with Application to Blood Testing
Source: PLoS One. 2012 Jan 19;7(1):e29059. doi: 10.1371/journal.pone.0029059 (PMC3261845; doi:10.1371/journal.pone.0029059)
Supplement: Table S5 — Discovery Probesets hypothesized to be switched-off in colorectal neoplastic tissues relative to non-neoplastic tissues. (DOC) [file pone.0029059.s005.doc]

**SUPPLEMENTARY TABLE S5.** Discovery Probesets hypothesized to be switched-off in colorectal neoplastic tissues relative to non-neoplastic tissues.

SUPPLEMENTAL TABLE S5

| Probeset | ID | Symbol |
| --- | --- | --- |
| 204719_at | ABCA8 | ATP-binding cass., sub-fam A (ABC1), 8 |
| 209613_s_at | ADH1B | alcohol dehydrogenase 1B (class I), beta polypeptide |
| 230788_at | GCNT2 | glycosaminyl (N-acetyl) transferase 2, I-branching enzyme (I blood group) |
| 228885_at | MAMDC2 | MAM domain containing 2 |
| 206637_at | P2RY14 | purinergic receptor P2Y, G-protein coupled, 14 |
| 204931_at | TCF21 | trancription factor 21 |
| 228504_at | -NA- | -NA- |
| 225575_at | LIFR | leukemia inhibitory factor receptor alpha |
| 231925_at | P2RY1 | purinergic receptor P2Y, G-protein coupled, 1 |
| 207980_s_at | CITED2 | Cbp/p300-interacting transactivator, with Glu/Asp-rich carboxy-terminal domain, 2 |
| 227827_at | SORBS2 | sorbin and SH3 domain containing 2 |
| 209170_s_at | GPM6B | glycoprotein M6B |
| 220376_at | LRRC19 | leucine rich repeat containing 19 |
| 231773_at | ANGPTL1 | angiopoietin-like 1 |
| 207080_s_at | PYY | peptide YY |
| 235146_at | -NA- | -NA- |
| 228706_s_at | CLDN23 | claudin 23 |
| 231120_x_at | PKIB | protein kianse (cAMP-dependent, catalytic) inhibitor beta |
| 202920_at | ANK2 | ankyrin 2, neuronal |
| 211549_s_at | HPGD | hydroxyprostaglandin dehydrogenase 15-(NAD) |
| 228854_at | -NA- | -NA- |
| 224412_s_at | TRPM6 | transient receptor potential cation channel, subfamily M, member 6 |
| 220812_s_at | HHLA2 | HERV-H LTR-associating 2 |
| 220037_s_at | LYVE1 | lymphatic vessel endothelial hyaluronan receptor 1 |
| 222717_at | SDPR | serum deprivation response (phosphatidyl-serine binding protein) |
| 205433_at | BCHE | butyrylcholinesterase |
| 203296_s_at | ATP1A2 | ATPase, Na+/K+ transporting alpha 2 (+) polypeptide |
| 219948_x_at | UGT2A3 | UDP glucuronosyltransferase 2 family, polypeptide A3 |
| 228766_at | CD36 | CD36 molecule (thrombospondin receptor) |
| 243278_at | FOXP2 | Forkhead box P2 |
| 203881_s_at | DMD | dystrophin (muscular dystrophy, Duchenne and Becker types) |
| 204940_at | PLN | phospholamban |
| 206664_at | SI | sucrase-isomaltase (alpha-glucosidase) |
| 214598_at | CLDN8 | claudin 8 |
| 238751_at | SORBS2 | sorbin and SH3 containing 2 |
