# Supplementary material for: Discovery and Validation of Molecular Biomarkers for Colorectal Adenomas and Cancer with Application to Blood Testing
Source: PLoS One. 2012 Jan 19;7(1):e29059. doi: 10.1371/journal.pone.0029059 (PMC3261845; doi:10.1371/journal.pone.0029059)
Supplement: Table S6 — Confidence intervals of sensitivity and specificity for each validated up-regulated probeset in colorectal neoplasia (19 adenomas + 19 cancers) relative to 30 normal colon tissue specimens (validation data). Note that sensitivity and specificity calculations are estimated based from the mid-point of ROC curves (approximate inflection point) and are included for comparison purposes only. (DOC) [file pone.0029059.s006.doc]

**SUPPLEMENTARY TABLE S6.** Confidence intervals of sensitivity and specificity for each validated up-regulated probeset in colorectal neoplasia (19 adenomas + 19 cancers) relative to 30 normal colon tissue specimens (validation data). Note that sensitivity and specificity calculations are estimated based from the mid-point of ROC curves (approximate inflection point) and are included for comparison purposes only.

SUPPLEMENTAL TABLE S6

| Discovery Probeset | Symbol | Probe  sets Tested | Mean Diff (P<=0.5) | FC>=2.0 | Fold Change | t-Value | Adj. P Value | Best Probeset | Sens/Spec |
| --- | --- | --- | --- | --- | --- | --- | --- | --- | --- |
| 203256_at | CDH3 | 23 | + | + | 37.54 | -20.93 | 3.75E-31 | 802708-HuGene_st | 98.31 (95.5-99.5) |
| 200660_at | S100A11 | 24 | + | + | 3.83 | -17.82 | 2.68E-27 | 1050447-HuGene_st | 97.42 (93.9-99.1) |
| 201341_at | ENC1 | 24 | + | + | 3.93 | -16.47 | 1.44E-26 | 216704-HuGene_st | 96.67 (92.6-98.7) |
| 202431_s_at | MYC | 25 | + | + | 4.11 | -16.38 | 5.19E-25 | 292645-HuGene_st | 96.41 (92.2-98.6) |
| 221577_x_at | GDF15 | 25 | + | + | 5.84 | -16.09 | 5.51E-25 | 716715-HuGene_st | 96.23 (91.9-98.5) |
| 203962_s_at | NEBL | 30 | + | + | 5.52 | -16.19 | 5.41E-24 | 923272-HuGene_st | 96.13 (91.8-98.4) |
| 203961_at | NEBL | 30 | + | + | 5.52 | -16.19 | 5.41E-24 | 923272-HuGene_st | 96.11 (91.8-98.4) |
| 204702_s_at | NFE2L3 | 15 | + | + | 4.57 | -16.12 | 1.51E-24 | 95873-HuGene_st | 96.09 (91.7-98.4) |
| 201563_at | SORD | 24 | + | + | 4.36 | -15.91 | 3.44E-25 | 510024-HuGene_st | 95.85 (91.3-98.3) |
| 207850_at | CXCL3 | 22 | + | + | 7.85 | -14.92 | 5.76E-22 | 11327-HuGene_st | 95.01 (90-97.8) |
| 209774_x_at | CXCL2 | 65 | + | + | 7.85 | -14.86 | 1.88E-21 | 11327-HuGene_st | 95 (90-97.8) |
| 204259_at | MMP7 | 23 | + | + | 69.29 | -14.81 | 7.61E-22 | 267137-HuGene_st | 94.86 (89.8-97.7) |
| 222549_at | CLDN1 | 47 | + | + | 13.09 | -14.76 | 6.10E-22 | 557209-HuGene_st | 94.81 (89.7-97.7) |
| 228754_at | SLC6A6 | 28 | + | + | 5.02 | -14.58 | 1.15E-21 | 128406-HuGene_st | 94.7 (89.5-97.6) |
| 218872_at | TESC | 24 | + | + | 16.84 | -14.50 | 3.26E-21 | 921201-HuGene_st | 94.6 (89.4-97.6) |
| 219911_s_at | SLCO4A1 | 25 | + | + | 4.84 | -13.87 | 1.46E-20 | 181673-HuGene_st | 93.92 (88.4-97.2) |
| 202935_s_at | SOX9 | 24 | + | + | 3.85 | -13.84 | 1.71E-20 | 772629-HuGene_st | 93.91 (88.4-97.2) |
| 202936_s_at | SOX9 | 23 | + | + | 3.85 | -13.84 | 1.63E-20 | 772629-HuGene_st | 93.91 (88.4-97.2) |
| 203510_at | MET | 26 | + | + | 3.35 | -13.52 | 1.50E-20 | 388394-HuGene_st | 93.83 (88.3-97.1) |
| 212942_s_at | *KIAA1199* | 27 | + | + | 25.16 | -13.77 | 8.12E-21 | 1008852-HuGene_st | 93.65 (88-97) |
| 209369_at | ANXA3 | 23 | + | + | 3.34 | -13.16 | 1.15E-20 | 839382-HuGene_st | 93.6 (87.9-97) |
| 204404_at | SLC12A2 | 24 | + | + | 3.42 | -13.36 | 1.40E-20 | 636328-HuGene_st | 93.5 (87.8-96.9) |
| 225835_at | SLC12A2 | 24 | + | + | 3.42 | -13.36 | 1.40E-20 | 636328-HuGene_st | 93.49 (87.8-96.9) |
| 201417_at | SOX4 | 28 | + | + | 2.89 | -13.46 | 2.30E-20 | 455510-HuGene_st | 93.45 (87.8-96.9) |
| 201416_at | SOX4 | 28 | + | + | 2.89 | -13.46 | 2.30E-20 | 455510-HuGene_st | 93.43 (87.8-96.9) |
| 204351_at | S100P | 23 | + | + | 4.37 | -12.86 | 4.56E-19 | 28680-HuGene_st | 92.97 (87-96.6) |
| 227475_at | FOXQ1 | 23 | + | + | 10.97 | -13.11 | 4.85E-19 | 763699-HuGene_st | 92.96 (87.1-96.6) |
| 212531_at | LCN2 | 23 | + | + | 14.60 | -13.02 | 5.68E-19 | 769185-HuGene_st | 92.91 (87-96.6) |
| 204470_at | CXCL1 | 23 | + | + | 10.40 | -13.03 | 5.53E-19 | 448247-HuGene_st | 92.8 (86.9-96.5) |
| 218704_at | RNF43 | 50 | + | + | 3.24 | -12.83 | 2.12E-18 | 680908-HuGene_st | 92.8 (86.8-96.5) |
| 208712_at | CCND1 | 24 | + | + | 2.85 | -12.83 | 7.06E-20 | 210671-HuGene_st | 92.71 (86.7-96.4) |
| 205983_at | DPEP1 | 23 | + | + | 23.90 | -12.93 | 5.27E-19 | 195459-HuGene_st | 92.56 (86.6-96.3) |
| 202504_at | TRIM29 | 25 | + | + | 11.01 | -12.51 | 2.36E-18 | 84230-HuGene_st | 92.09 (85.8-96) |
| 229215_at | ASCL2 | 24 | + | + | 7.73 | -12.20 | 7.76E-18 | 342676-HuGene_st | 91.61 (85.2-95.7) |
| 201195_s_at | SLC7A5 | 23 | + | + | 6.31 | -12.18 | 5.11E-18 | 496060-HuGene_st | 91.58 (85.2-95.7) |
| 217996_at | PHLDA1 | 28 | + | + | 5.27 | -11.95 | 1.34E-17 | 810737-HuGene_st | 91.13 (84.7-95.4) |
| 201506_at | TGFBI | 23 | + | + | 4.31 | -11.67 | 4.14E-17 | 374147-HuGene_st | 91.04 (84.5-95.3) |
| 205476_at | CCL20 | 23 | + | + | 10.14 | -11.69 | 2.03E-17 | 454699-HuGene_st | 90.5 (83.8-94.9) |
| 218984_at | PUS7 | 23 | + | + | 3.06 | -11.23 | 5.26E-16 | 188478-HuGene_st | 90.34 (83.5-94.8) |
| 225541_at | RPL22L1 | 45 | + | + | 4.19 | -11.34 | 4.37E-16 | 772808-HuGene_st | 90.31 (83.6-94.8) |
| 212070_at | GPR56 | 24 | + | + | 2.60 | -11.25 | 7.45E-17 | 463959-HuGene_st | 90.23 (83.5-94.8) |
| 212281_s_at | TMEM97 | 25 | + | + | 2.77 | -11.23 | 1.88E-16 | 458988-HuGene_st | 90.17 (83.4-94.7) |
| 210511_s_at | INHBA | 25 | + | + | 3.76 | -10.90 | 2.80E-15 | 402595-HuGene_st | 89.56 (82.6-94.3) |
| 223062_s_at | PSAT1 | 17 | + | + | 5.52 | -10.77 | 3.32E-15 | 139416-HuGene_st | 89.32 (82.3-94.1) |
| 225520_at | MTHFD1L | 28 | + | + | 4.04 | -10.83 | 4.46E-15 | 478940-HuGene_st | 89.3 (82.3-94.1) |
| 222696_at | AXIN2 | 26 | + | + | 4.44 | -10.55 | 1.90E-15 | 466765-HuGene_st | 88.81 (81.7-93.7) |
| 201666_at | TIMP1 | 23 | + | + | 3.01 | -10.31 | 2.44E-15 | 676809-HuGene_st | 88.78 (81.6-93.7) |
| 219787_s_at | ECT2 | 25 | + | + | 2.82 | -10.37 | 1.42E-14 | 770888-HuGene_st | 88.75 (81.6-93.7) |
| 206224_at | CST1 | 9 | + | + | 13.01 | -10.47 | 7.04E-15 | 95123-HuGene_st | 88.65 (81.5-93.7) |
| 209309_at | AZGP1 | 23 | + | + | 6.19 | -10.42 | 1.31E-14 | 363333-HuGene_st | 88.46 (81.2-93.5) |
| 202286_s_at | TACSTD2 | 26 | + | + | 15.71 | -10.26 | 3.13E-14 | 1004611-HuGene_st | 88.17 (80.9-93.3) |
| 218507_at | HIG2 | 25 | + | + | 4.25 | -10.30 | 2.50E-14 | 246364-HuGene_st | 88.17 (80.8-93.3) |
| 205513_at | TCN1 | 23 | + | + | 15.22 | -9.94 | 4.81E-14 | 293164-HuGene_st | 87.51 (80-92.8) |
| 200832_s_at | SCD | 18 | + | + | 4.28 | -9.77 | 1.23E-13 | 446192-HuGene_st | 87.25 (79.7-92.6) |
| 224915_x_at | C20orf199 | 26 | + | + | 2.87 | -9.79 | 8.45E-14 | 323199-HuGene_st | 87.22 (79.7-92.6) |
| 226835_s_at | C20orf199 | 26 | + | + | 2.87 | -9.79 | 8.45E-14 | 323199-HuGene_st | 87.21 (79.7-92.6) |
| 224428_s_at | CDCA7 | 24 | + | + | 4.27 | -9.84 | 6.41E-14 | 776822-HuGene_st | 87.12 (79.6-92.5) |
| 204170_s_at | CKS2 | 15 | + | + | 4.15 | -9.67 | 2.13E-13 | 139489-HuGene_st | 86.88 (79.3-92.3) |
| 210766_s_at | CSE1L | 26 | + | + | 2.28 | -9.51 | 7.98E-13 | 757480-HuGene_st | 86.79 (79.2-92.2) |
| 204855_at | SERPINB5 | 24 | + | + | 10.36 | -9.10 | 4.80E-12 | 237063-HuGene_st | 85.61 (77.8-91.4) |
| 206286_s_at | TDGF1 | 24 | + | + | 6.33 | -9.17 | 3.07E-12 | 509506-HuGene_st | 85.53 (77.8-91.3) |
| 203878_s_at | MMP11 | 27 | + | + | 4.55 | -9.04 | 6.30E-12 | 324921-HuGene_st | 85.25 (77.4-91.1) |
| 213880_at | LGR5 | 25 | + | + | 7.89 | -8.84 | 1.10E-11 | 784585-HuGene_st | 84.91 (76.9-90.8) |
| 222449_at | TMEPAI | 26 | + | + | 2.20 | -8.51 | 3.73E-11 | 69901-HuGene_st | 84.88 (76.9-90.8) |
| 227174_at | WDR72 | 25 | + | + | 14.11 | -8.53 | 5.22E-11 | 829740-HuGene_st | 83.95 (75.9-90.1) |
| 60474_at | FERMT1 | 1 | + | + | 2.28 | -8.06 | 7.37E-16 | 60474_at | 83.32 (75.2-89.6) |
| 205828_at | MMP3 | 23 | + | + | 15.63 | -8.08 | 1.44E-10 | 542596-HuGene_st | 82.83 (74.6-89.2) |
| 218963_s_at | KRT23 | 23 | + | + | 6.81 | -8.04 | 3.58E-10 | 962342-HuGene_st | 82.74 (74.4-89.1) |
| 204475_at | MMP1 | 23 | + | + | 10.59 | -7.75 | 1.22E-09 | 61706-HuGene_st | 81.79 (73.3-88.4) |
| 204580_at | MMP12 | 23 | + | + | 6.24 | -7.58 | 1.99E-09 | 372865-HuGene_st | 81.43 (73-88.1) |
| 222608_s_at | ANLN | 24 | + | + | 2.63 | -7.39 | 4.94E-09 | 522941-HuGene_st | 80.96 (72.5-87.7) |
| 210052_s_at | TPX2 | 23 | + | + | 2.03 | -6.52 | 1.56E-07 | 210052_s_at | 79.77 (71.1-86.7) |
| 202954_at | UBE2C | 23 | + | + | 2.82 | -6.98 | 1.74E-08 | 352222-HuGene_st | 79.48 (70.8-86.5) |
| 225664_at | COL12A1 | 49 | + | + | 3.16 | -6.91 | 9.48E-08 | 405565-HuGene_st | 79.34 (70.6-86.4) |
| 205890_s_at | UBD | 23 | + | + | 6.59 | -6.93 | 4.07E-08 | 918426-HuGene_st | 79.32 (70.6-86.4) |
| 211506_s_at | IL8 | 90 | + | + | 3.10 | -6.88 | 1.99E-07 | 497219-HuGene_st | 79.31 (70.6-86.4) |
| 202859_x_at | IL8 | 90 | + | + | 3.10 | -6.88 | 1.99E-07 | 497219-HuGene_st | 79.29 (70.5-86.3) |
| 213905_x_at | BGN | 25 | + | + | 2.69 | -6.72 | 1.01E-07 | 279145-HuGene_st | 78.75 (69.9-85.8) |
| 37892_at | COL11A1 | 48 | + | + | 2.60 | -6.63 | 2.93E-07 | 254341-HuGene_st | 78.36 (69.6-85.6) |
| 207457_s_at | LY6G6D | 23 | + | + | 6.64 | -6.62 | 1.34E-07 | 80885-HuGene_st | 78.17 (69.3-85.4) |
| 232252_at | DUSP27 | 23 | + | + | 4.81 | -6.35 | 4.68E-07 | 612025-HuGene_st | 77.51 (68.7-84.8) |
| 225681_at | CTHRC1 | 23 | + | + | 2.57 | -5.43 | 1.83E-05 | 235232-HuGene_st | 74.1 (64.9-81.9) |
| 214974_x_at | CXCL5 | 25 | + | + | 4.48 | -5.35 | 2.79E-05 | 264137-HuGene_st | 73.9 (64.7-81.7) |
| 207173_x_at | CDH11 | 27 | + | + | 2.25 | -5.31 | 3.44E-05 | 167614-HuGene_st | 73.63 (64.5-81.4) |
| 209955_s_at | FAP | 45 | + | + | 2.33 | -5.24 | 6.66E-05 | 993707-HuGene_st | 73.48 (64.3-81.3) |
| 205815_at | REG3A | 18 | + | + | 12.09 | -5.10 | 2.22E-05 | 290546-HuGene_st | 72.96 (63.7-80.9) |
| 205886_at | REG1B | 22 | + | + | 13.15 | -4.92 | 8.08E-05 | 796972-HuGene_st | 72.08 (62.9-80.2) |
| 209218_at | SQLE | 26 | + | + | 2.69 | -4.76 | 1.21E-04 | 181699-HuGene_st | 71.51 (62.2-79.5) |
| 209875_s_at | SPP1 | 24 | + | + | 3.32 | -4.46 | 4.55E-04 | 1070547-HuGene_st | 70.33 (60.9-78.5) |
| 202310_s_at | COL1A1 | 27 | + | + | 2.50 | -4.23 | 1.82E-03 | 487433-HuGene_st | 69.25 (59.8-77.6) |
| 202311_s_at | COL1A1 | 27 | + | + | 2.50 | -4.23 | 1.82E-03 | 487433-HuGene_st | 69.25 (59.8-77.5) |
| 209752_at | REG1A | 23 | + | + | 10.93 | -4.18 | 6.19E-04 | 657765-HuGene_st | 69.23 (59.8-77.5) |
| 225295_at | SLC39A10 | 25 | + | - | 1.93 | -8.69 | 2.32E-11 | 238968_at | 85.55 (77.6-91.3) |
| 218796_at | FERMT1 | 1 | + | - | 1.96 | -7.77 | 8.05E-15 | 218796_at | 82.46 (74.1-88.9) |
| 212353_at | SULF1 | 26 | + | - | 1.74 | -7.32 | 9.70E-09 | 807443-HuGene_st | 81.3 (72.9-87.9) |
| 212344_at | SULF1 | 26 | + | - | 1.74 | -7.32 | 9.70E-09 | 807443-HuGene_st | 81.3 (72.7-88) |
| 212354_at | SULF1 | 26 | + | - | 1.74 | -7.32 | 9.70E-09 | 807443-HuGene_st | 81.29 (72.9-88) |
| 225806_at | JUB | 1 | + | - | 1.19 | -6.84 | 8.08E-12 | 225806_at | 79.55 (70.8-86.5) |
| 204885_s_at | MSLN | 23 | + | - | 1.93 | -5.83 | 3.78E-06 | 63302-HuGene_st | 76.22 (67.2-83.7) |
| 232151_at | 7A5 | 1 | + | - | 1.52 | -5.77 | 7.79E-09 | 232151_at | 75.85 (66.8-83.4) |
| 241031_at | FAM148A | 1 | + | - | 1.25 | -5.29 | 1.24E-07 | 241031_at | 74.02 (64.8-81.8) |
| 212190_at | SERPINE2 | 25 | + | - | 1.90 | -4.66 | 2.23E-04 | 443903-HuGene_st | 71.36 (62-79.5) |
| 226237_at | COL8A1 | 26 | + | - | 1.43 | -3.45 | 2.55E-02 | 713455-HuGene_st | 66.14 (56.6-74.7) |
| 204051_s_at | SFRP4 | 24 | - | - | 1.29 | -2.83 | 8.29E-02 | 1011118-HuGene_st | 63.67 (54.1-72.4) |
| 202404_s_at | COL1A2 | 25 | - | - | 1.90 | -2.75 | 1.89E-01 | 240155-HuGene_st | 62.75 (53.2-71.6) |
| 238021_s_at | hCG_1815491 | 1 | - | - | 1.07 | -1.92 | 5.48E-02 | 238021_s_at | 59.36 (49.8-68.4) |
| 227140_at | NA | 1 | - | - | 1.04 | -1.28 | 2.01E-01 | 227140_at | 56.29 (46.7-65.5) |
| 203083_at | THBS2 | 23 | - | - | 1.26 | -1.15 | 9.84E-01 | 565405-HuGene_st | 55.54 (46-64.8) |
