# Supplementary material for: Discovery and Validation of Molecular Biomarkers for Colorectal Adenomas and Cancer with Application to Blood Testing
Source: PLoS One. 2012 Jan 19;7(1):e29059. doi: 10.1371/journal.pone.0029059 (PMC3261845; doi:10.1371/journal.pone.0029059)
Supplement: Table S7 — Confidence intervals of sensitivity and specificity for each validated down-regulated probeset target in colorectal neoplasia (19 adenomas + 19 cancers) relative to 30 normal colon tissue specimens. Note that sensitivity and specificity calculations are estimated based from the mid-point of ROC curves (approximate inflection point) and are included for comparison purposes only. (DOC) [file pone.0029059.s007.doc]

**SUPPLEMENTARY TABLE S7.** Confidence intervals of sensitivity and specificity for each validated down-regulated probeset target in colorectal neoplasia (19 adenomas + 19 cancers) relative to 30 normal colon tissue specimens. Note that sensitivity and specificity calculations are estimated based from the mid-point of ROC curves (approximate inflection point) and are included for comparison purposes only.

SUPPLEMENTAL TABLE S7

| Discovery Probeset | Symbol | Probe  sets Tested | Mean Diff (P<=0.5) | FC>=2 | Fold Change | t-Value | Adj. P Value | Best Probeset | Sens/Spec |
| --- | --- | --- | --- | --- | --- | --- | --- | --- | --- |
| 230788_at | GCNT2 | 28 | + | + | 13.36 | 18.74 | 2.16E-27 | 935239-HuGene_st | 97.59 (94.2-99.2) |
| 203908_at | SLC4A4 | 27 | + | + | 3.58 | 15.98 | 2.23E-23 | 810103-HuGene_st | 96.53 (92.4-98.6) |
| 206208_at | CA4 | 24 | + | + | 10.41 | 16.67 | 1.73E-24 | 636392-HuGene_st | 96.52 (92.4-98.6) |
| 207502_at | GUCA2B | 23 | + | + | 51.78 | 16.87 | 7.04E-25 | 636392-HuGene_st | 96.51 (92.4-98.6) |
| 206209_s_at | CA4 | 24 | + | + | 10.41 | 16.67 | 1.73E-24 | 276512-HuGene_st | 96.51 (92.4-98.6) |
| 207003_at | GUCA1B | 25 | + | + | 10.70 | 16.62 | 7.32E-25 | 207003_at | 96.25 (92-98.5) |
| 205950_s_at | CA1 | 24 | + | + | 38.27 | 15.22 | 3.15E-22 | 632246-HuGene_st | 95.4 (90.6-98) |
| 206784_at | AQP8 | 23 | + | + | 10.82 | 14.61 | 2.98E-22 | 486167-HuGene_st | 95.21 (90.3-97.9) |
| 220834_at | MS4A12 | 23 | + | + | 7.15 | 12.98 | 2.13E-19 | 220834_at | 95.02 (90-97.8) |
| 209735_at | ABCG2 | 23 | + | + | 31.53 | 14.74 | 1.09E-21 | 236197-HuGene_st | 94.85 (89.8-97.7) |
| 228195_at | MGC13057 | 24 | + | + | 3.18 | 14.14 | 6.94E-21 | 926449-HuGene_st | 94.54 (89.4-97.6) |
| 223754_at | MGC13057 | 24 | + | + | 3.18 | 14.14 | 6.94E-21 | 926449-HuGene_st | 94.54 (89.3-97.5) |
| 220026_at | CLCA4 | 23 | + | + | 16.30 | 12.77 | 1.25E-19 | 220026_at | 94.48 (89.2-97.5) |
| 230830_at | OSTbeta | 23 | + | + | 7.15 | 14.28 | 6.23E-21 | 552746-HuGene_st | 94.44 (89.2-97.5) |
| 231120_x_at | PKIB | 24 | + | + | 3.28 | 13.76 | 5.94E-20 | 866170-HuGene_st | 94.09 (88.7-97.3) |
| 223551_at | PKIB | 24 | + | + | 3.28 | 13.76 | 5.94E-20 | 866170-HuGene_st | 94.07 (88.6-97.3) |
| 226492_at | SEMA6D | 27 | + | + | 4.27 | 13.69 | 6.07E-20 | 855766-HuGene_st | 93.9 (88.4-97.2) |
| 228707_at | CLDN23 | 25 | + | + | 3.47 | 13.68 | 4.27E-20 | 403960-HuGene_st | 93.8 (88.2-97.1) |
| 228706_s_at | CLDN23 | 25 | + | + | 3.47 | 13.68 | 4.27E-20 | 403960-HuGene_st | 93.79 (88.3-97.1) |
| 224836_at | TP53INP2 | 23 | + | + | 3.78 | 13.39 | 1.45E-19 | 224836_at | 93.49 (87.8-96.9) |
| 209612_s_at | ADH1B | 25 | + | + | 4.67 | 13.10 | 4.70E-19 | 1078343-HuGene_st | 93.44 (87.8-96.9) |
| 209613_s_at | ADH1B | 25 | + | + | 4.67 | 13.10 | 4.70E-19 | 1078343-HuGene_st | 93.42 (87.7-96.9) |
| 224412_s_at | TRPM6 | 26 | + | + | 7.50 | 13.24 | 2.25E-19 | 767074-HuGene_st | 93.38 (87.7-96.9) |
| 209301_at | CA2 | 23 | + | + | 9.22 | 13.05 | 5.87E-19 | 762141-HuGene_st | 93.1 (87.3-96.7) |
| 208399_s_at | EDN3 | 24 | + | + | 11.21 | 13.05 | 6.42E-19 | 314103-HuGene_st | 92.91 (86.9-96.5) |
| 215657_at | SLC26A3 | 24 | + | + | 21.40 | 12.15 | 4.18E-18 | 206143_at | 92.79 (86.9-96.5) |
| 206143_at | SLC26A3 | 24 | + | + | 21.40 | 12.15 | 4.18E-18 | 206143_at | 92.79 (86.8-96.5) |
| 228961_at | MIER3 | 28 | + | + | 3.91 | 12.72 | 9.14E-19 | 884917-HuGene_st | 92.77 (86.9-96.5) |
| 204719_at | ABCA8 | 25 | + | + | 6.82 | 12.86 | 1.26E-18 | 57305-HuGene_st | 92.76 (86.8-96.5) |
| 231975_s_at | MIER3 | 28 | + | + | 3.91 | 12.72 | 9.14E-19 | 884917-HuGene_st | 92.76 (86.9-96.4) |
| 218756_s_at | MGC4172 | 23 | + | + | 3.74 | 12.69 | 1.74E-18 | 339712-HuGene_st | 92.68 (86.7-96.4) |
| 207977_s_at | DPT | 25 | + | + | 9.07 | 12.81 | 1.53E-18 | 876245-HuGene_st | 92.65 (86.6-96.4) |
| 213068_at | DPT | 25 | + | + | 9.07 | 12.81 | 1.53E-18 | 876245-HuGene_st | 92.64 (86.7-96.4) |
| 213071_at | DPT | 25 | + | + | 9.07 | 12.81 | 1.53E-18 | 876245-HuGene_st | 92.62 (86.6-96.3) |
| 204036_at | EDG2 | 27 | + | + | 3.19 | 12.64 | 2.44E-18 | 816816-HuGene_st | 92.55 (86.6-96.3) |
| 202037_s_at | SFRP1 | 26 | + | + | 15.53 | 12.69 | 2.77E-18 | 722739-HuGene_st | 92.41 (86.3-96.2) |
| 211548_s_at | HPGD | 27 | + | + | 3.21 | 11.98 | 3.65E-17 | 291863-HuGene_st | 92.27 (86.1-96.1) |
| 211549_s_at | HPGD | 27 | + | + | 3.21 | 11.98 | 3.65E-17 | 291863-HuGene_st | 92.27 (86.2-96.1) |
| 203913_s_at | HPGD | 27 | + | + | 3.21 | 11.98 | 3.65E-17 | 291863-HuGene_st | 92.27 (86.1-96.1) |
| 203914_x_at | HPGD | 27 | + | + | 3.21 | 11.98 | 3.65E-17 | 291863-HuGene_st | 92.24 (86.2-96.1) |
| 206710_s_at | EPB41L3 | 25 | + | + | 2.51 | 12.07 | 2.62E-17 | 275431-HuGene_st | 92.13 (86-96) |
| 214142_at | ZG16 | 23 | + | + | 8.14 | 11.96 | 2.51E-17 | 287680-HuGene_st | 91.99 (85.7-96) |
| 231773_at | ANGPTL1 | 47 | + | + | 4.64 | 12.35 | 1.42E-17 | 572942-HuGene_st | 91.89 (85.6-95.9) |
| 205480_s_at | UGP2 | 25 | + | + | 2.41 | 11.75 | 1.64E-17 | 795967-HuGene_st | 91.85 (85.6-95.9) |
| 206198_s_at | CEACAM7 | 69 | + | + | 10.40 | 12.27 | 3.96E-17 | 206199_at | 91.83 (85.5-95.8) |
| 206199_at | CEACAM7 | 69 | + | + | 10.40 | 12.27 | 3.96E-17 | 206199_at | 91.82 (85.6-95.8) |
| 211848_s_at | CEACAM7 | 69 | + | + | 10.40 | 12.27 | 3.96E-17 | 206199_at | 91.82 (85.5-95.9) |
| 225575_at | LIFR | 30 | + | + | 6.36 | 12.22 | 2.45E-17 | 275506-HuGene_st | 91.8 (85.5-95.8) |
| 204955_at | SRPX | 23 | + | + | 3.91 | 12.14 | 1.79E-17 | 195599-HuGene_st | 91.75 (85.4-95.8) |
| 224823_at | MYLK | 27 | + | + | 3.99 | 12.10 | 2.33E-17 | 527465-HuGene_st | 91.71 (85.4-95.8) |
| 202555_s_at | MYLK | 27 | + | + | 3.99 | 12.10 | 2.33E-17 | 527465-HuGene_st | 91.7 (85.4-95.8) |
| 205112_at | PLCE1 | 30 | + | + | 2.43 | 11.90 | 6.91E-17 | 1562826_at | 91.6 (85.2-95.7) |
| 205464_at | SCNN1B | 23 | + | + | 16.15 | 11.98 | 3.20E-17 | 773390-HuGene_st | 91.33 (84.9-95.5) |
| 202992_at | C7 | 25 | + | + | 6.67 | 11.87 | 4.40E-17 | 893214-HuGene_st | 91.29 (84.9-95.5) |
| 222722_at | OGN | 24 | + | + | 13.67 | 11.92 | 2.25E-17 | 955964-HuGene_st | 91.2 (84.7-95.4) |
| 226430_at | RELL1 | 24 | + | + | 2.50 | 11.78 | 4.10E-17 | 753672-HuGene_st | 91.15 (84.7-95.4) |
| 229839_at | SCARA5 | 26 | + | + | 2.86 | 11.69 | 1.25E-16 | 217628_at | 91.11 (84.6-95.3) |
| 213317_at | CLIC5 | 27 | + | + | 2.37 | 11.32 | 3.68E-16 | 734536-HuGene_st | 91.11 (84.6-95.3) |
| 235849_at | SCARA5 | 26 | + | + | 2.86 | 11.69 | 1.25E-16 | 734536-HuGene_st | 91.07 (84.6-95.3) |
| 220812_s_at | HHLA2 | 25 | + | + | 10.02 | 11.71 | 3.47E-17 | 371335-HuGene_st | 90.93 (84.4-95.3) |
| 202242_at | TSPAN7 | 25 | + | + | 3.68 | 11.49 | 2.13E-16 | 703402-HuGene_st | 90.75 (84.2-95.1) |
| 202920_at | ANK2 | 28 | + | + | 6.76 | 11.59 | 2.59E-16 | 314086-HuGene_st | 90.74 (84.1-95.1) |
| 225275_at | EDIL3 | 28 | + | + | 2.96 | 11.33 | 6.21E-16 | 436107-HuGene_st | 90.54 (83.9-95) |
| 209074_s_at | FAM107A | 24 | + | + | 2.92 | 11.44 | 2.52E-16 | 315531-HuGene_st | 90.46 (83.8-94.9) |
| 203001_s_at | STMN2 | 24 | + | + | 6.13 | 11.46 | 2.25E-16 | 459270-HuGene_st | 90.44 (83.7-94.9) |
| 203000_at | STMN2 | 24 | + | + | 6.13 | 11.46 | 2.25E-16 | 459270-HuGene_st | 90.44 (83.7-94.9) |
| 204697_s_at | CHGA | 23 | + | + | 7.21 | 11.34 | 5.54E-16 | 152790-HuGene_st | 90.39 (83.7-94.9) |
| 209763_at | CHRDL1 | 23 | + | + | 13.88 | 11.44 | 1.49E-16 | 335657-HuGene_st | 90.39 (83.7-94.9) |
| 210946_at | PPAP2A | 26 | + | + | 2.40 | 11.13 | 6.43E-16 | 282276-HuGene_st | 90.3 (83.5-94.8) |
| 205259_at | NR3C2 | 26 | + | + | 3.19 | 11.25 | 2.08E-16 | 748063-HuGene_st | 90.15 (83.4-94.7) |
| 236313_at | CDKN2B | 24 | + | + | 10.09 | 11.11 | 1.34E-15 | 252481-HuGene_st | 89.82 (83-94.5) |
| 231925_at | P2RY1 | 24 | + | + | 3.60 | 11.01 | 1.41E-15 | 628249-HuGene_st | 89.74 (82.8-94.4) |
| 233565_s_at | SDCBP2 | 23 | + | + | 2.37 | 10.54 | 9.28E-15 | 233565_s_at | 89.32 (82.3-94.1) |
| 207080_s_at | PYY | 23 | + | + | 11.71 | 10.83 | 3.50E-15 | 656845-HuGene_st | 89.26 (82.2-94.1) |
| 223952_x_at | DHRS9 | 47 | + | + | 9.28 | 10.71 | 7.43E-15 | 413956-HuGene_st | 89.19 (82.2-94) |
| 219799_s_at | DHRS9 | 47 | + | + | 9.28 | 10.71 | 7.43E-15 | 413956-HuGene_st | 89.18 (82.1-94) |
| 224009_x_at | DHRS9 | 47 | + | + | 9.28 | 10.71 | 7.43E-15 | 413956-HuGene_st | 89.16 (82.2-94) |
| 202731_at | PDCD4 | 27 | + | + | 2.60 | 10.44 | 8.67E-16 | 681770-HuGene_st | 89.02 (82-93.9) |
| 205200_at | CLEC3B | 23 | + | + | 4.46 | 10.69 | 4.65E-15 | 794375-HuGene_st | 88.91 (81.7-93.8) |
| 209687_at | CXCL12 | 24 | + | + | 3.79 | 10.49 | 1.54E-14 | 567389-HuGene_st | 88.79 (81.7-93.7) |
| 219014_at | PLAC8 | 24 | + | + | 3.87 | 10.40 | 5.76E-15 | 219014_at | 88.74 (81.6-93.7) |
| 224959_at | SLC26A2 | 25 | + | + | 7.83 | 10.36 | 2.08E-14 | 322199-HuGene_st | 88.73 (81.5-93.7) |
| 215299_x_at | SULT1A1 | 20 | + | + | 3.37 | 10.54 | 7.17E-15 | 224963_at | 88.71 (81.5-93.7) |
| 205097_at | SLC26A2 | 25 | + | + | 7.83 | 10.36 | 2.08E-14 | 224963_at | 88.7 (81.6-93.7) |
| 224963_at | SLC26A2 | 25 | + | + | 7.83 | 10.36 | 2.08E-14 | 224963_at | 88.69 (81.5-93.7) |
| 218546_at | C1orf115 | 24 | + | + | 2.95 | 10.40 | 1.95E-14 | 293810-HuGene_st | 88.59 (81.3-93.6) |
| 242317_at | HIGD1A | 37 | + | + | 2.09 | 10.09 | 6.04E-14 | 936261-HuGene_st | 88.49 (81.3-93.5) |
| 221896_s_at | HIGD1A | 36 | + | + | 2.09 | 10.00 | 6.29E-14 | 936261-HuGene_st | 88.49 (81.3-93.5) |
| 206262_at | ADH1C | 22 | + | + | 3.10 | 9.55 | 2.94E-13 | 661427-HuGene_st | 88.21 (81-93.4) |
| 214091_s_at | GPX3 | 24 | + | + | 2.57 | 9.99 | 5.68E-14 | 623237-HuGene_st | 88.1 (80.8-93.3) |
| 201348_at | GPX3 | 24 | + | + | 2.57 | 9.99 | 5.68E-14 | 623237-HuGene_st | 88.09 (80.8-93.2) |
| 206149_at | CHP2 | 1 | + | + | 3.20 | 9.95 | 2.58E-23 | 206149_at | 88.03 (80.8-93.2) |
| 220037_s_at | LYVE1 | 1 | + | + | 2.76 | 9.93 | 3.07E-23 | 220037_s_at | 87.98 (80.6-93.2) |
| 208383_s_at | PCK1 | 23 | + | + | 3.60 | 9.47 | 5.74E-13 | 208383_s_at | 87.75 (80.4-93) |
| 211896_s_at | DCN | 30 | + | + | 5.62 | 9.95 | 2.05E-13 | 6640-HuGene_st | 87.63 (80.2-92.9) |
| 204931_at | TCF21 | 24 | + | + | 2.34 | 9.75 | 2.51E-13 | 804657-HuGene_st | 87.53 (80.1-92.8) |
| 220075_s_at | MUPCDH | 1 | + | + | 3.06 | 9.57 | 1.09E-21 | 220075_s_at | 87.18 (79.8-92.6) |
| 206134_at | ADAMDEC1 | 23 | + | + | 3.19 | 9.41 | 9.04E-13 | 893574-HuGene_st | 87.07 (79.6-92.5) |
| 205433_at | BCHE | 23 | + | + | 6.21 | 9.74 | 3.18E-13 | 228090-HuGene_st | 87.01 (79.5-92.4) |
| 206385_s_at | ANK3 | 28 | + | + | 2.45 | 9.57 | 7.46E-13 | 472262-HuGene_st | 86.79 (79.2-92.3) |
| 223395_at | ABI3BP | 25 | + | + | 3.62 | 9.50 | 9.47E-13 | 801423-HuGene_st | 86.65 (79.1-92.2) |
| 204818_at | HSD17B2 | 23 | + | + | 4.76 | 9.39 | 2.32E-13 | 281748-HuGene_st | 86.39 (78.7-91.9) |
| 226594_at | ENTPD5 | 26 | + | + | 3.01 | 9.26 | 1.43E-12 | 219796_s_at | 86.1 (78.4-91.8) |
| 219796_s_at | MUPCDH | 1 | + | + | 2.52 | 9.12 | 7.85E-20 | 760608-HuGene_st | 86.09 (78.4-91.7) |
| 209373_at | MALL | 23 | + | + | 3.96 | 9.22 | 2.10E-12 | 209373_at | 85.99 (78.2-91.7) |
| 209167_at | GPM6B | 50 | + | + | 3.59 | 9.29 | 4.04E-12 | 599862-HuGene_st | 85.96 (78.2-91.7) |
| 209170_s_at | GPM6B | 50 | + | + | 3.59 | 9.29 | 4.04E-12 | 599862-HuGene_st | 85.95 (78.2-91.6) |
| 205593_s_at | PDE9A | 69 | + | + | 3.91 | 9.25 | 6.56E-12 | 779446-HuGene_st | 85.85 (78.1-91.6) |
| 202291_s_at | MGP | 24 | + | + | 4.68 | 9.15 | 3.63E-12 | 696658-HuGene_st | 85.8 (78-91.5) |
| 201739_at | SGK1 | 1 | + | + | 2.90 | 8.97 | 2.90E-19 | 201739_at | 85.77 (77.9-91.5) |
| 206422_at | GCG | 23 | + | + | 12.56 | 9.06 | 5.58E-12 | 1044997-HuGene_st | 85.51 (77.6-91.3) |
| 225720_at | SYNPO2 | 28 | + | + | 6.43 | 9.06 | 5.07E-12 | 800385-HuGene_st | 85.41 (77.6-91.2) |
| 225721_at | SYNPO2 | 28 | + | + | 6.43 | 9.06 | 5.07E-12 | 800385-HuGene_st | 85.4 (77.5-91.2) |
| 227662_at | SYNPO2 | 28 | + | + | 6.43 | 9.06 | 5.07E-12 | 800385-HuGene_st | 85.4 (77.5-91.2) |
| 225895_at | SYNPO2 | 28 | + | + | 6.43 | 9.06 | 5.07E-12 | 800385-HuGene_st | 85.38 (77.5-91.2) |
| 225894_at | SYNPO2 | 28 | + | + | 6.43 | 9.06 | 5.07E-12 | 800385-HuGene_st | 85.37 (77.5-91.2) |
| 209668_x_at | CES2 | 25 | + | + | 2.87 | 8.98 | 3.51E-12 | 817786-HuGene_st | 85.33 (77.4-91.2) |
| 209667_at | CES2 | 25 | + | + | 2.87 | 8.98 | 3.51E-12 | 11827-HuGene_st | 85.3 (77.5-91.1) |
| 213953_at | KRT20 | 23 | + | + | 5.49 | 9.08 | 1.26E-12 | 817786-HuGene_st | 85.25 (77.4-91.1) |
| 204130_at | HSD11B2 | 23 | + | + | 2.53 | 8.87 | 6.97E-12 | 78582-HuGene_st | 85.2 (77.4-91.1) |
| 219508_at | GCNT3 | 24 | + | + | 3.94 | 8.86 | 8.39E-12 | 230376_at | 85.16 (77.3-91) |
| 220376_at | LRRC19 | 23 | + | + | 4.51 | 8.76 | 6.75E-12 | 177641-HuGene_st | 85.01 (77.1-90.9) |
| 215125_s_at | UGT1A6 | 24 | + | + | 4.56 | 8.84 | 1.02E-11 | 116025-HuGene_st | 84.96 (77-90.9) |
| 207126_x_at | UGT1A1 | 23 | + | + | 4.56 | 8.84 | 9.98E-12 | 116025-HuGene_st | 84.95 (77-90.9) |
| 204532_x_at | UGT1A9 | 27 | + | + | 4.56 | 8.84 | 1.18E-11 | 116025-HuGene_st | 84.94 (77.1-90.9) |
| 208596_s_at | UGT1A3 | 23 | + | + | 4.56 | 8.84 | 1.01E-11 | 116025-HuGene_st | 84.93 (76.9-90.9) |
| 219669_at | CD177 | 20 | + | + | 5.77 | 8.73 | 1.49E-11 | 993284-HuGene_st | 84.87 (76.9-90.8) |
| 219059_s_at | LYVE1 | 1 | + | + | 2.15 | 8.58 | 9.11E-18 | 219059_s_at | 84.77 (76.8-90.7) |
| 209498_at | CEACAM1 | 27 | + | + | 2.85 | 8.63 | 2.66E-11 | 209498_at | 84.71 (76.8-90.6) |
| 206576_s_at | CEACAM1 | 27 | + | + | 2.85 | 8.63 | 2.66E-11 | 72610-HuGene_st | 84.69 (76.7-90.7) |
| 210298_x_at | FHL1 | 27 | + | + | 3.25 | 8.74 | 1.63E-11 | 72610-HuGene_st | 84.69 (76.7-90.6) |
| 201539_s_at | FHL1 | 27 | + | + | 3.25 | 8.74 | 1.63E-11 | 72610-HuGene_st | 84.68 (76.7-90.7) |
| 201540_at | FHL1 | 27 | + | + | 3.25 | 8.74 | 1.63E-11 | 209498_at | 84.68 (76.7-90.6) |
| 214505_s_at | FHL1 | 27 | + | + | 3.25 | 8.74 | 1.63E-11 | 209498_at | 84.68 (76.6-90.7) |
| 210299_s_at | FHL1 | 27 | + | + | 3.25 | 8.74 | 1.63E-11 | 72610-HuGene_st | 84.66 (76.7-90.6) |
| 211889_x_at | CEACAM1 | 27 | + | + | 2.85 | 8.63 | 2.66E-11 | 72610-HuGene_st | 84.65 (76.7-90.7) |
| 201427_s_at | SEPP1 | 27 | + | + | 4.15 | 8.67 | 2.83E-11 | 803943-HuGene_st | 84.56 (76.5-90.5) |
| 204388_s_at | MAOA | 26 | + | + | 2.18 | 7.89 | 2.49E-10 | 234534_at | 84.48 (76.5-90.5) |
| 204389_at | MAOA | 26 | + | + | 2.18 | 7.89 | 2.49E-10 | 234534_at | 84.46 (76.4-90.5) |
| 212741_at | MAOA | 26 | + | + | 2.18 | 7.89 | 2.49E-10 | 234534_at | 84.45 (76.4-90.5) |
| 209791_at | PADI2 | 25 | + | + | 3.98 | 8.63 | 1.46E-11 | 391522-HuGene_st | 84.44 (76.4-90.5) |
| 202995_s_at | FBLN1 | 27 | + | + | 3.55 | 8.62 | 2.82E-11 | 546550-HuGene_st | 84.39 (76.4-90.4) |
| 203881_s_at | DMD | 26 | + | + | 4.74 | 8.59 | 5.01E-11 | 945600-HuGene_st | 84.36 (76.3-90.4) |
| 203963_at | CA12 | 29 | + | + | 2.65 | 8.51 | 4.32E-11 | 89078-HuGene_st | 84.19 (76.1-90.3) |
| 214164_x_at | CA12 | 29 | + | + | 2.65 | 8.51 | 4.32E-11 | 89078-HuGene_st | 84.19 (76.1-90.3) |
| 210735_s_at | CA12 | 29 | + | + | 2.65 | 8.51 | 4.32E-11 | 89078-HuGene_st | 84.17 (76.1-90.3) |
| 204508_s_at | CA12 | 29 | + | + | 2.65 | 8.51 | 4.32E-11 | 89078-HuGene_st | 84.17 (76.1-90.3) |
| 215867_x_at | CA12 | 29 | + | + | 2.65 | 8.51 | 4.32E-11 | 89078-HuGene_st | 84.16 (76.2-90.3) |
| 206000_at | MEP1A | 23 | + | + | 3.17 | 8.06 | 2.07E-10 | 854525-HuGene_st | 84.02 (75.9-90.1) |
| 205892_s_at | FABP1 | 25 | + | + | 6.54 | 8.37 | 9.02E-11 | 825235-HuGene_st | 83.76 (75.6-89.9) |
| 203343_at | UGDH | 44 | + | + | 2.40 | 8.29 | 8.72E-11 | 961600-HuGene_st | 83.67 (75.5-89.9) |
| 226304_at | HSPB6 | 24 | + | + | 6.88 | 8.37 | 6.16E-11 | 438264-HuGene_st | 83.56 (75.4-89.8) |
| 206561_s_at | AKR1B10 | 13 | + | + | 5.17 | 8.19 | 7.91E-11 | 1017995-HuGene_st | 83.5 (75.4-89.7) |
| 236300_at | NA | 1 | + | + | 2.13 | 8.11 | 5.14E-16 | 236300_at | 83.44 (75.3-89.7) |
| 213624_at | SMPDL3A | 24 | + | + | 3.14 | 8.24 | 5.84E-11 | 734740-HuGene_st | 83.39 (75.2-89.7) |
| 235146_at | NA | 1 | + | + | 2.09 | 7.97 | 1.60E-15 | 235146_at | 83.08 (74.8-89.4) |
| 238143_at | LOC646627 | 1 | + | + | 5.02 | 7.97 | 1.58E-15 | 238143_at | 83.04 (74.8-89.3) |
| 214598_at | CLDN8 | 23 | + | + | 12.96 | 7.93 | 2.92E-10 | 1018006-HuGene_st | 82.42 (74.1-88.9) |
| 201496_x_at | MYH11 | 29 | + | + | 5.35 | 7.95 | 2.15E-10 | 628303-HuGene_st | 82.37 (74-88.8) |
| 207961_x_at | MYH11 | 29 | + | + | 5.35 | 7.95 | 2.15E-10 | 628303-HuGene_st | 82.37 (74.1-88.8) |
| 201497_x_at | MYH11 | 29 | + | + | 5.35 | 7.95 | 2.15E-10 | 628303-HuGene_st | 82.36 (74-88.8) |
| 201495_x_at | MYH11 | 29 | + | + | 5.35 | 7.95 | 2.15E-10 | 628303-HuGene_st | 82.33 (74.1-88.8) |
| 221841_s_at | KLF4 | 24 | + | + | 2.27 | 7.68 | 1.19E-09 | 310345-HuGene_st | 82.28 (73.9-88.7) |
| 220266_s_at | KLF4 | 24 | + | + | 2.27 | 7.68 | 1.19E-09 | 310345-HuGene_st | 82.28 (73.9-88.7) |
| 220468_at | ARL14 | 23 | + | + | 3.06 | 7.75 | 7.01E-10 | 220468_at | 81.91 (73.4-88.5) |
| 204834_at | FGL2 | 24 | + | + | 2.07 | 7.53 | 2.64E-09 | 884418-HuGene_st | 81.91 (73.6-88.5) |
| 227265_at | FGL2 | 24 | + | + | 2.07 | 7.53 | 2.64E-09 | 884418-HuGene_st | 81.91 (73.5-88.5) |
| 223484_at | C15orf48 | 23 | + | + | 3.07 | 7.69 | 1.54E-09 | 661133-HuGene_st | 81.83 (73.4-88.4) |
| 217897_at | FXYD6 | 24 | + | + | 2.92 | 7.78 | 6.39E-10 | 138547-HuGene_st | 81.7 (73.3-88.3) |
| 225458_at | EXOC3 | 26 | + | + | 3.17 | 7.65 | 2.25E-09 | 225458_at | 81.52 (73.1-88.2) |
| 209114_at | TSPAN1 | 23 | + | + | 2.16 | 7.46 | 1.16E-09 | 833585-HuGene_st | 81.46 (73-88) |
| 210302_s_at | MAB21L2 | 24 | + | + | 5.20 | 7.63 | 1.05E-09 | 338297-HuGene_st | 81.24 (72.7-87.9) |
| 209283_at | CRYAB | 23 | + | + | 3.91 | 7.58 | 1.47E-09 | 581894-HuGene_st | 81.17 (72.6-87.8) |
| 202888_s_at | ANPEP | 25 | + | + | 8.18 | 7.47 | 2.04E-09 | 42832-HuGene_st | 81.15 (72.6-87.8) |
| 213921_at | SST | 23 | + | + | 6.14 | 7.48 | 4.16E-09 | 477599-HuGene_st | 81.11 (72.6-87.8) |
| 222162_s_at | ADAMTS1 | 24 | + | + | 2.37 | 7.37 | 6.42E-09 | 432578-HuGene_st | 80.88 (72.3-87.6) |
| 221748_s_at | TNS1 | 50 | + | + | 2.01 | 7.25 | 2.21E-08 | 149647-HuGene_st | 80.81 (72.2-87.5) |
| 219948_x_at | UGT2A3 | 23 | + | + | 4.89 | 7.32 | 8.64E-09 | 766751-HuGene_st | 80.8 (72.3-87.5) |
| 221747_at | TNS1 | 50 | + | + | 2.01 | 7.25 | 2.21E-08 | 766751-HuGene_st | 80.76 (72.2-87.5) |
| 214027_x_at | DES | 47 | + | + | 6.66 | 7.34 | 1.47E-08 | 680953-HuGene_st | 80.58 (72-87.4) |
| 202222_s_at | DES | 47 | + | + | 6.66 | 7.34 | 1.47E-08 | 680953-HuGene_st | 80.56 (72-87.4) |
| 202742_s_at | PRKACB | 25 | + | + | 2.18 | 7.21 | 1.30E-08 | 202741_at | 80.56 (71.9-87.3) |
| 207432_at | BEST2 | 23 | + | + | 3.63 | 7.28 | 9.19E-09 | 202741_at | 80.53 (72-87.3) |
| 202741_at | PRKACB | 25 | + | + | 2.18 | 7.21 | 1.30E-08 | 242542-HuGene_st | 80.52 (72-87.3) |
| 230087_at | PRIMA1 | 23 | + | + | 2.75 | 7.26 | 4.74E-09 | 83688-HuGene_st | 80.31 (71.7-87.2) |
| 225207_at | PDK4 | 25 | + | + | 3.02 | 7.19 | 1.45E-08 | 1084922-HuGene_st | 80.31 (71.7-87.2) |
| 221004_s_at | ITM2C | 23 | + | + | 2.22 | 7.28 | 3.87E-09 | 806272-HuGene_st | 80.31 (71.7-87.2) |
| 203980_at | FABP4 | 24 | + | + | 5.55 | 7.11 | 2.01E-08 | 547124-HuGene_st | 79.93 (71.3-86.8) |
| 239272_at | MMP28 | 27 | + | + | 2.47 | 7.04 | 1.80E-08 | 578497-HuGene_st | 79.71 (71.1-86.6) |
| 242601_at | LOC253012 | 23 | + | + | 4.46 | 6.90 | 3.59E-08 | 873053-HuGene_st | 79.56 (70.8-86.6) |
| 205382_s_at | CFD | 23 | + | + | 2.81 | 6.99 | 3.06E-08 | 396618-HuGene_st | 79.49 (70.8-86.5) |
| 217546_at | MT1M | 101 | + | + | 2.41 | 6.88 | 2.14E-07 | 723245-HuGene_st | 79.48 (70.8-86.5) |
| 200795_at | SPARCL1 | 23 | + | + | 2.85 | 6.84 | 1.33E-08 | 246509-HuGene_st | 79.35 (70.7-86.3) |
| 226303_at | PGM5 | 23 | + | + | 5.87 | 6.85 | 5.87E-08 | 810480-HuGene_st | 79.14 (70.4-86.1) |
| 227006_at | PPP1R14A | 23 | + | + | 2.51 | 6.86 | 3.54E-08 | 374630-HuGene_st | 79.02 (70.3-86.1) |
| 208763_s_at | TSC22D3 | 25 | + | + | 2.76 | 6.80 | 7.22E-08 | 235364_at | 79 (70.3-86.1) |
| 229070_at | C6orf105 | 24 | + | + | 2.13 | 6.47 | 1.38E-07 | 669403-HuGene_st | 78.89 (70.2-85.9) |
| 212730_at | DMN | 24 | + | + | 4.05 | 6.78 | 4.36E-08 | 207872-HuGene_st | 78.88 (70.2-86) |
| 222717_at | SDPR | 24 | + | + | 2.21 | 6.73 | 9.62E-08 | 878908-HuGene_st | 78.81 (70.1-85.9) |
| 203766_s_at | LMOD1 | 25 | + | + | 2.97 | 6.64 | 5.55E-08 | 611871-HuGene_st | 78.52 (69.7-85.6) |
| 215118_s_at | IGHA1 | 103 | + | + | 3.00 | 6.56 | 2.69E-07 | 1044209-HuGene_st | 78.24 (69.4-85.4) |
| 217022_s_at | IGHA1 | 103 | + | + | 3.00 | 6.56 | 2.69E-07 | 1044209-HuGene_st | 78.23 (69.4-85.4) |
| 204894_s_at | AOC3 | 19 | + | + | 2.92 | 6.60 | 6.43E-08 | 557248-HuGene_st | 78.16 (69.3-85.4) |
| 228202_at | PLN | 26 | + | + | 2.01 | 6.41 | 4.16E-07 | 204938_s_at | 78.01 (69.2-85.2) |
| 204940_at | PLN | 25 | + | + | 2.01 | 6.40 | 4.14E-07 | 204938_s_at | 78 (69.2-85.2) |
| 204938_s_at | PLN | 25 | + | + | 2.01 | 6.40 | 4.14E-07 | 204938_s_at | 77.98 (69.1-85.2) |
| 204939_s_at | PLN | 25 | + | + | 2.01 | 6.40 | 4.14E-07 | 204938_s_at | 77.97 (69.1-85.2) |
| 205403_at | IL1R2 | 24 | + | + | 2.54 | 6.45 | 1.73E-07 | 377084-HuGene_st | 77.69 (68.8-84.9) |
| 211372_s_at | IL1R2 | 24 | + | + | 2.54 | 6.45 | 1.73E-07 | 377084-HuGene_st | 77.69 (68.8-84.9) |
| 221667_s_at | HSPB8 | 17 | + | + | 3.85 | 6.40 | 2.61E-07 | 688528-HuGene_st | 77.51 (68.6-84.8) |
| 202350_s_at | MATN2 | 24 | + | + | 2.23 | 6.33 | 2.11E-07 | 902099-HuGene_st | 77.44 (68.5-84.8) |
| 221584_s_at | KCNMA1 | 27 | + | + | 2.37 | 6.29 | 6.86E-07 | 554163-HuGene_st | 77.28 (68.4-84.6) |
| 202746_at | ITM2A | 24 | + | + | 2.77 | 6.32 | 5.27E-07 | 391687-HuGene_st | 77.27 (68.4-84.6) |
| 207245_at | UGT2B17 | 9 | + | + | 4.88 | 6.19 | 3.75E-07 | 357315-HuGene_st | 77.04 (68.1-84.4) |
| 225782_at | MSRB3 | 29 | + | + | 2.24 | 6.18 | 1.20E-06 | 483115-HuGene_st | 76.96 (68-84.3) |
| 241994_at | XDH | 24 | + | + | 2.03 | 6.13 | 8.13E-07 | 919455-HuGene_st | 76.94 (67.9-84.3) |
| 226654_at | MUC12 | 26 | + | + | 4.89 | 6.41 | 1.53E-07 | 412725-HuGene_st | 76.64 (67.7-84.1) |
| 208581_x_at | MT1X | 18 | + | + | 4.35 | 6.09 | 9.84E-07 | 788358-HuGene_st | 76.43 (67.5-83.9) |
| 204326_x_at | MT1X | 18 | + | + | 4.35 | 6.09 | 9.84E-07 | 788358-HuGene_st | 76.4 (67.4-83.9) |
| 203951_at | CNN1 | 23 | + | + | 4.56 | 6.08 | 4.77E-07 | 337187-HuGene_st | 76.31 (67.3-83.8) |
| 218087_s_at | SORBS1 | 29 | + | + | 2.54 | 6.01 | 2.18E-06 | 1041567-HuGene_st | 76.23 (67.2-83.7) |
| 223623_at | C2orf40 | 23 | + | + | 2.97 | 6.01 | 1.69E-06 | 1011921-HuGene_st | 76.08 (67-83.5) |
| 201957_at | PPP1R12B | 29 | + | + | 2.21 | 5.75 | 4.26E-06 | 23543-HuGene_st | 75.14 (66.1-82.8) |
| 207392_x_at | UGT2B15 | 25 | + | + | 5.72 | 5.62 | 9.73E-06 | 500573-HuGene_st | 74.98 (65.9-82.6) |
| 206377_at | FOXF2 | 23 | + | + | 2.08 | 5.48 | 9.50E-06 | 589126-HuGene_st | 74.3 (65.1-82.1) |
| 238751_at | SORBS2 | 56 | + | + | 2.10 | 5.45 | 4.23E-05 | 238751_at | 74.28 (65.1-82) |
| 227826_s_at | SORBS2 | 56 | + | + | 2.10 | 5.45 | 4.23E-05 | 238751_at | 74.27 (65.1-81.9) |
| 227827_at | SORBS2 | 56 | + | + | 2.10 | 5.45 | 4.23E-05 | 238751_at | 74.25 (65.1-81.9) |
| 225728_at | SORBS2 | 56 | + | + | 2.10 | 5.45 | 4.23E-05 | 238751_at | 74.24 (65.1-82) |
| 227727_at | MRGPRF | 23 | + | + | 2.99 | 5.47 | 1.52E-05 | 20487-HuGene_st | 74.21 (65.1-81.9) |
| 209656_s_at | TMEM47 | 24 | + | + | 2.02 | 5.43 | 1.86E-05 | 717973-HuGene_st | 74.17 (65.1-82) |
| 228232_s_at | VSIG2 | 24 | + | + | 2.05 | 5.41 | 2.01E-05 | 985113-HuGene_st | 74.15 (65-81.9) |
| 209948_at | KCNMB1 | 24 | + | + | 2.66 | 5.13 | 3.74E-05 | 17348-HuGene_st | 72.87 (63.7-80.8) |
| 206461_x_at | MT1H | 37 | + | + | 4.55 | 5.01 | 1.51E-04 | 197989-HuGene_st | 72.39 (63.1-80.3) |
| 220645_at | FAM55D | 23 | + | + | 3.48 | 4.97 | 5.67E-05 | 633727-HuGene_st | 72.23 (62.9-80.2) |
| 201058_s_at | MYL9 | 25 | + | + | 2.18 | 4.70 | 3.18E-04 | 201058_s_at | 71.37 (62.1-79.4) |
| 204745_x_at | MT1G | 21 | + | + | 2.66 | 4.61 | 2.58E-04 | 226585-HuGene_st | 70.71 (61.4-78.9) |
| 205547_s_at | TAGLN | 26 | + | + | 2.12 | 4.54 | 3.46E-04 | 279405-HuGene_st | 70.71 (61.4-78.9) |
| 228133_s_at | NDE1 | 28 | + | + | 2.02 | 4.63 | 2.26E-05 | 228133_s_at | 70.63 (61.3-78.8) |
| 208450_at | LGALS2 | 23 | + | + | 3.07 | 4.57 | 4.64E-04 | 204754-HuGene_st | 70.62 (61.3-78.8) |
| 206641_at | TNFRSF17 | 23 | + | + | 2.08 | 4.24 | 1.55E-03 | 1062688-HuGene_st | 69.49 (60.1-77.8) |
| 212592_at | IGJ | 1 | + | + | 2.58 | 4.10 | 4.21E-05 | 212592_at | 69.23 (59.8-77.6) |
| 217165_x_at | MT1F | 23 | + | + | 2.20 | 4.04 | 1.20E-03 | 987574-HuGene_st | 68.56 (59.1-76.9) |
| 213629_x_at | MT1F | 23 | + | + | 2.20 | 4.04 | 1.20E-03 | 987574-HuGene_st | 68.56 (59.1-76.9) |
| 206664_at | SI | 23 | + | + | 2.79 | 3.87 | 5.48E-03 | 9605-HuGene_st | 67.89 (58.4-76.4) |
| 204607_at | HMGCS2 | 23 | + | + | 2.67 | 3.74 | 2.28E-03 | 729816-HuGene_st | 66.97 (57.4-75.5) |
| 223597_at | ITLN1 | 23 | + | + | 2.54 | 2.99 | 2.10E-02 | 389198-HuGene_st | 63.93 (54.4-72.7) |
| 207761_s_at | METTL7A | 25 | + | - | 1.93 | 12.81 | 1.35E-18 | 278779-HuGene_st | 94.08 (88.6-97.3) |
| 228766_at | CD36 | 27 | + | - | 1.65 | 10.11 | 6.27E-14 | 392196-HuGene_st | 89.14 (82.2-94) |
| 209357_at | CITED2 | 25 | + | - | 1.64 | 9.47 | 5.29E-13 | 125201-HuGene_st | 87.19 (79.7-92.6) |
| 207980_s_at | CITED2 | 25 | + | - | 1.64 | 9.47 | 5.29E-13 | 125201-HuGene_st | 87.18 (79.7-92.6) |
| 204034_at | ETHE1 | 23 | + | - | 1.75 | 9.10 | 3.46E-12 | 1008653-HuGene_st | 86.58 (78.9-92.1) |
| 224480_s_at | AGPAT9 | 1 | + | - | 1.57 | 8.54 | 1.31E-17 | 224480_s_at | 84.62 (76.6-90.6) |
| 206637_at | P2RY14 | 23 | + | - | 2.00 | 8.30 | 1.31E-10 | 528057-HuGene_st | 83.93 (75.8-90) |
| 212814_at | AHCYL2 | 1 | + | - | 1.76 | 7.99 | 1.35E-15 | 212814_at | 83.11 (75-89.4) |
| 228469_at | PPID | 25 | + | - | 1.73 | 7.53 | 3.34E-09 | 204185_x_at | 81.65 (73.2-88.2) |
| 201324_at | EMP1 | 26 | + | - | 1.95 | 7.05 | 2.59E-08 | 201324_at | 80.46 (71.9-87.3) |
| 226818_at | MPEG1 | 20 | + | - | 1.86 | 6.84 | 4.58E-08 | 226818_at | 79.49 (70.7-86.5) |
| 203060_s_at | PAPSS2 | 26 | + | - | 1.97 | 6.77 | 6.43E-08 | 1040734-HuGene_st | 79.09 (70.4-86.1) |
| 212288_at | FNBP1 | 112 | + | - | 1.55 | 6.62 | 3.54E-08 | 728822-HuGene_st | 78.78 (70.1-85.9) |
| 217967_s_at | FAM129A | 24 | + | - | 1.80 | 5.70 | 4.59E-06 | 148319-HuGene_st | 76 (67-83.5) |
| 226333_at | NA | 1 | + | - | 1.16 | 5.79 | 6.94E-09 | 226333_at | 75.97 (66.9-83.4) |
| 202274_at | ACTG2 | 24 | + | - | 1.82 | 5.57 | 1.07E-05 | 667818-HuGene_st | 75.81 (66.7-83.4) |
| 227522_at | CMBL | 25 | + | - | 1.58 | 5.58 | 1.09E-05 | 157156-HuGene_st | 75.16 (66-82.8) |
| 226302_at | ATP8B1 | 23 | + | - | 1.67 | 5.48 | 5.73E-06 | 1088077-HuGene_st | 74.39 (65.3-82.1) |
| 212192_at | KCTD12 | 24 | + | - | 1.86 | 5.24 | 1.96E-05 | 509699-HuGene_st | 73.44 (64.2-81.3) |
| 208792_s_at | CLU | 26 | + | - | 1.42 | 4.89 | 1.61E-04 | 222043_at | 73.1 (63.9-81) |
| 208791_at | CLU | 26 | + | - | 1.42 | 4.89 | 1.61E-04 | 222043_at | 73.09 (63.9-81) |
| 228885_at | MAMDC2 | 1 | + | - | 1.65 | 4.92 | 8.50E-07 | 228885_at | 72.6 (63.3-80.6) |
| 205935_at | FOXF1 | 23 | + | - | 1.28 | 4.57 | 4.46E-04 | 21663-HuGene_st | 72.16 (62.9-80.2) |
| 202388_at | RGS2 | 23 | + | - | 1.94 | 4.82 | 1.71E-04 | 331021-HuGene_st | 71.95 (62.8-80) |
| 224989_at | NA | 1 | + | - | 1.75 | 4.59 | 4.52E-06 | 224989_at | 71.28 (61.9-79.4) |
| 224663_s_at | CFL2 | 25 | + | - | 1.75 | 4.39 | 9.84E-04 | 545956-HuGene_st | 70.16 (60.9-78.4) |
| 224352_s_at | CFL2 | 25 | + | - | 1.75 | 4.39 | 9.84E-04 | 545956-HuGene_st | 70.15 (60.8-78.4) |
| 214433_s_at | SELENBP1 | 25 | + | - | 1.70 | 4.33 | 9.08E-04 | 914892-HuGene_st | 70.1 (60.8-78.3) |
| 212859_x_at | MT1E | 1 | + | - | 1.78 | 4.20 | 2.70E-05 | 212859_x_at | 69.66 (60.2-77.9) |
| 212097_at | CAV1 | 22 | + | - | 1.58 | 4.20 | 1.67E-03 | 793412-HuGene_st | 69.52 (60.1-77.7) |
| 211644_x_at | HLA-C | 71 | + | - | 1.24 | 3.74 | 2.68E-02 | 754900-HuGene_st | 67.8 (58.3-76.3) |
| 214768_x_at | HLA-C | 71 | + | - | 1.24 | 3.74 | 2.68E-02 | 754900-HuGene_st | 67.79 (58.3-76.3) |
| 211643_x_at | HLA-C | 71 | + | - | 1.24 | 3.74 | 2.68E-02 | 754900-HuGene_st | 67.78 (58.2-76.3) |
| 226811_at | FAM46C | 24 | + | - | 1.38 | 3.75 | 8.57E-03 | 197110-HuGene_st | 67.67 (58.1-76.1) |
| 214696_at | C17orf91 | 1 | + | - | 1.17 | 3.55 | 3.79E-04 | 214696_at | 66.85 (57.4-75.4) |
| 205554_s_at | DNASE1L3 | 24 | + | - | 1.52 | 3.55 | 8.41E-03 | 867148-HuGene_st | 66.65 (57.2-75.2) |
| 217109_at | MUC4 | 50 | + | - | 1.65 | 3.58 | 6.35E-03 | 1061780-HuGene_st | 66.62 (57.1-75.2) |
| 217110_s_at | MUC4 | 50 | + | - | 1.65 | 3.58 | 6.35E-03 | 1061780-HuGene_st | 66.61 (57.1-75.2) |
| 204895_x_at | MUC4 | 50 | + | - | 1.65 | 3.58 | 6.35E-03 | 1061780-HuGene_st | 66.6 (57.1-75.2) |
| 204897_at | PTGER4 | 24 | + | - | 1.41 | 3.41 | 2.38E-02 | 450915-HuGene_st | 65.75 (56.2-74.4) |
| 213746_s_at | FLNA | 25 | + | - | 1.59 | 3.27 | 1.58E-02 | 296693-HuGene_st | 65.48 (55.9-74.1) |
| 203240_at | FCGBP | 23 | + | - | 1.99 | 3.43 | 1.22E-02 | 22847-HuGene_st | 65.26 (55.8-73.9) |
| 210524_x_at | NA | 1 | + | - | 1.40 | 3.17 | 1.54E-03 | 210524_x_at | 65.15 (55.6-73.8) |
| 204083_s_at | TPM2 | 25 | + | - | 1.53 | 3.08 | 2.60E-02 | 828216-HuGene_st | 64.68 (55.1-73.4) |
| 229659_s_at | PIGR | 25 | + | - | 1.59 | 3.08 | 2.78E-02 | 173623-HuGene_st | 64.21 (54.7-72.9) |
| 227735_s_at | C10orf99 | 24 | + | - | 1.76 | 3.00 | 2.19E-02 | 748831-HuGene_st | 64.19 (54.6-72.9) |
| 214916_x_at | IL8 | 91 | + | - | 1.46 | 2.99 | 1.48E-02 | 227735_s_at | 64.17 (54.6-72.9) |
| 227736_at | C10orf99 | 24 | + | - | 1.76 | 3.00 | 2.19E-02 | 227735_s_at | 64.16 (54.6-72.9) |
| 200621_at | CSRP1 | 23 | + | - | 1.39 | 3.00 | 2.31E-02 | 453033-HuGene_st | 64.13 (54.5-72.8) |
| 209210_s_at | FERMT2 | 1 | + | - | 1.11 | 2.01 | 4.43E-02 | 209210_s_at | 59.78 (50.2-68.8) |
| 209374_s_at | IGHM | 56 | - | - | 1.19 | 2.88 | 7.31E-02 | 212827_at | 66.41 (56.8-75) |
| 216491_x_at | IGHM | 56 | - | - | 1.19 | 2.88 | 7.31E-02 | 212827_at | 66.35 (56.9-75) |
| 216510_x_at | ZCWPW2 | 64 | - | - | 1.36 | 2.85 | 9.11E-02 | 1042953-HuGene_st | 63.58 (54-72.4) |
| 217148_x_at | IGL@ | 45 | - | - | 1.26 | 2.80 | 1.55E-01 | 38807-HuGene_st | 63.37 (53.8-72.2) |
| 205267_at | POU2AF1 | 25 | - | - | 1.18 | 2.67 | 2.39E-01 | 205267_at | 62.86 (53.3-71.7) |
| 202768_at | FOSB | 23 | - | - | 2.17 | 2.67 | 2.08E-01 | 739750-HuGene_st | 62.51 (53-71.4) |
| 217232_x_at | HBB | 21 | - | - | 1.17 | 2.05 | 4.62E-01 | 102408-HuGene_st | 60.19 (50.6-69.2) |
| 211696_x_at | HBB | 21 | - | - | 1.17 | 2.05 | 4.62E-01 | 102408-HuGene_st | 60.19 (50.6-69.1) |
| 209116_x_at | HBB | 21 | - | - | 1.17 | 2.05 | 4.62E-01 | 102408-HuGene_st | 60.17 (50.7-69.2) |
| 215176_x_at | NTN2L | 24 | - | - | 1.43 | 1.73 | 9.86E-01 | 679204-HuGene_st | 58.23 (48.7-67.4) |
| 216576_x_at | NTN2L | 24 | - | - | 1.43 | 1.73 | 9.86E-01 | 679204-HuGene_st | 58.23 (48.6-67.4) |
| 210107_at | CLCA1 | 23 | - | - | 1.66 | 1.59 | 4.16E-01 | 372283-HuGene_st | 57.65 (48.1-66.8) |
| 212224_at | ALDH1A1 | 23 | - | - | 1.40 | 1.49 | 4.23E-01 | 420405-HuGene_st | 57.02 (47.4-66.2) |
| 228854_at | NA | 1 | - | - | 1.05 | 1.30 | 1.93E-01 | 228854_at | 56.38 (46.8-65.6) |
| 217414_x_at | HBA1 | 27 | - | - | 1.11 | 1.04 | 9.98E-01 | 416795-HuGene_st | 55.51 (45.9-64.8) |
| 214414_x_at | HBA1 | 27 | - | - | 1.11 | 1.04 | 9.98E-01 | 416795-HuGene_st | 55.5 (45.9-64.8) |
| 209458_x_at | HBA1 | 27 | - | - | 1.11 | 1.04 | 9.98E-01 | 416795-HuGene_st | 55.5 (45.9-64.8) |
| 217378_x_at | NA | 1 | - | - | 1.15 | 1.12 | 2.63E-01 | 416795-HuGene_st | 55.49 (46-64.8) |
| 211745_x_at | HBA2 | 23 | - | - | 1.11 | 1.05 | 9.98E-01 | 217378_x_at | 55.49 (45.9-64.7) |
| 204018_x_at | HBA1 | 27 | - | - | 1.11 | 1.04 | 9.98E-01 | 416795-HuGene_st | 55.49 (45.9-64.7) |
| 211699_x_at | HBA1 | 27 | - | - | 1.11 | 1.04 | 9.98E-01 | 416795-HuGene_st | 55.48 (46-64.7) |
| 228504_at | NA | 1 | - | - | 1.04 | 1.08 | 2.79E-01 | 228504_at | 55.3 (45.7-64.5) |
| 214777_at | NA | 1 | - | - | 1.20 | 1.06 | 2.90E-01 | 214777_at | 55.18 (45.7-64.4) |
| 224342_x_at | LOC96610 | 1 | - | - | 1.15 | 1.05 | 2.95E-01 | 224342_x_at | 55.17 (45.6-64.5) |
| 234764_x_at | IGLV1-44 | 9 | - | - | 1.15 | 1.07 | 9.78E-01 | 224342_x_at | 55.14 (45.6-64.4) |
| 216984_x_at | RPL14 | 4 | - | - | 1.12 | 0.86 | 3.93E-01 | 216984_x_at | 54.11 (44.6-63.5) |
| 211645_x_at | NA | 1 | - | - | 1.13 | 0.80 | 4.23E-01 | 211645_x_at | 53.94 (44.4-63.3) |
| 217235_x_at | RPL14 | 4 | - | - | 1.11 | 0.81 | 4.22E-01 | 217235_x_at | 53.89 (44.3-63.2) |
| 216401_x_at | NA | 1 | - | - | 1.09 | 0.70 | 4.86E-01 | 216401_x_at | 53.44 (43.9-62.8) |
| 216207_x_at | IGKV1D-13 | 1 | - | - | 1.10 | 0.65 | 5.18E-01 | 216207_x_at | 53.17 (43.6-62.5) |
| 227725_at | ST6GALNAC1 | 23 | - | - | 1.06 | 0.47 | 9.45E-01 | 227725_at | 52.59 (43.1-62) |
| 217258_x_at | IVD | 22 | - | - | 1.04 | 0.42 | 6.78E-01 | 988503-HuGene_st | 51.99 (42.4-61.3) |
| 217179_x_at | LOC96610 | 1 | - | - | 1.04 | 0.29 | 7.73E-01 | 217179_x_at | 51.42 (41.9-60.9) |
| 228640_at | PCDH7 | 1 | - | - | 1.00 | -0.12 | 9.07E-01 | 228640_at | 50.57 (41.1-59.9) |
