# Supplementary material for: Discovery and Validation of Molecular Biomarkers for Colorectal Adenomas and Cancer with Application to Blood Testing
Source: PLoS One. 2012 Jan 19;7(1):e29059. doi: 10.1371/journal.pone.0029059 (PMC3261845; doi:10.1371/journal.pone.0029059)
Supplement: Supporting Information S1 — Quality Control Analysis. This document details the quality control methods applied to the discovery data to assess Gene Chip quality. (PDF) [file pone.0029059.s008.pdf]

CONFIDENTIAL

Quality Control Analysis of Gene Logic Data

Prepared for the Preventative Health Flagship
and CSIRO Mathematics and Information Sciences

by

Lawrence LaPointe

August 17, 2005

Technical Report Number: 05/205

CSIRO BIOINFORMATICS

Preventative Health

NATIONAL RESEARCH
FLAGSHIPS

CSIRO

CONTENTS

Description of Gene Logic data 3

Quality Control of Affymetrix Gene Chips 3

Scaling Factors 4

Background Values 4

Percent Present 5

Spike-in probesets 6

Control Probe Degradation 6

RNA Degradation Analysis 7

Within-probeset degradation 8

28S:18S Ratios 10

Principal Component Analysis 10

Scrubbed Array Summary 14

Code and Supplemental Material 14

References 14

Description of Gene Logic data

Gene expression and clinical descriptions for 548 colorectal tissue specimens were purchased from Gene Logic (Gaithersburg, MD, USA) to support the aims of the Preventative Health Flagship. Specifically, these data will be mined to identify biomarkers for specific colorectal tissue states and to better understand colorectal biology.

The Gene Logic data set was chosen after a comprehensive review of public and private data source options. These data will complement the Flagship's own efforts to produce high quality data.

For each of 548 tissues, CSIRO received:

- raw .CEL files produced by the Affymetrix (Santa Clara, CA, USA) Gene Chip® microarray system[1].
- Results from HG133A and HG133B chips, a total of 44,928 probesets
- more than 81 experimental and clinical descriptors for each tissue.

Quality Control of Affymetrix Gene Chips

Measuring tissue gene expression using high dimensional microarrays involves complex clinical and laboratory processing. The first step in analysing a set of expression arrays, therefore, should be a careful assessment of the data quality to identify, and possibly remove, potentially contaminating arrays from the analysis. This assessment includes basic editing and data review that is fundamental to any multivariate analysis [2].

Affymetrix data quality documentation recommends to focus on four data aspects for quality controlling batches of hybridised Gene Chips:[3]:

Absolute background (taken to be the lowest 2% of probe intensities)
Scale factor used to transform all probesets to absolute intensity of 100
Percentage of probesets (genes) 'called' present
Ratio of 3' to 5' binding for 'housekeeping' genes
Response of spike-in controls

To assess these qc parameters, we analysed the complete set of 548 chips using 'simpleaffy' [4] and 'affy' [5], BioConductor packages that provide convenient access to the Affymetrix qc metrics and normalisation algorithms. BioConductor is an open source R framework that provides a wide range of bioinformatics tools for analysing molecular biological data.[6][4]

Gene expression levels were calculated by both Microarray Suite (MAS) 5.0 (Affymetrix) and the Robust Multichip Average (RMA) normalisation techniques. [7] [8] [9]. Further, the data set was processed both as a single set of 44K probesets as well as by splitting the data into two sub arrays, the HG133A chip (22K probesets) and HG133B chip (22K probesets).

NOTE: The availability of two independently hybridised arrays for each tissue sample (ChipA and ChipB) provides a useful means to assess qc parameters in the Gene Logic data set. While the same hybridisation solution for a given tissue will be reacted with both chips, anomalous or outlier results at the tissue-hyb-solution level can be easily observed by inspection.

Scaling Factors

By default, the MAS5.0 normalisation algorithm sets the trimmed mean intensity of every array to an arbitrary level (target=100). The scaling factor is a measure of the scaling applied to each individual array to bring the average intensity to this value.

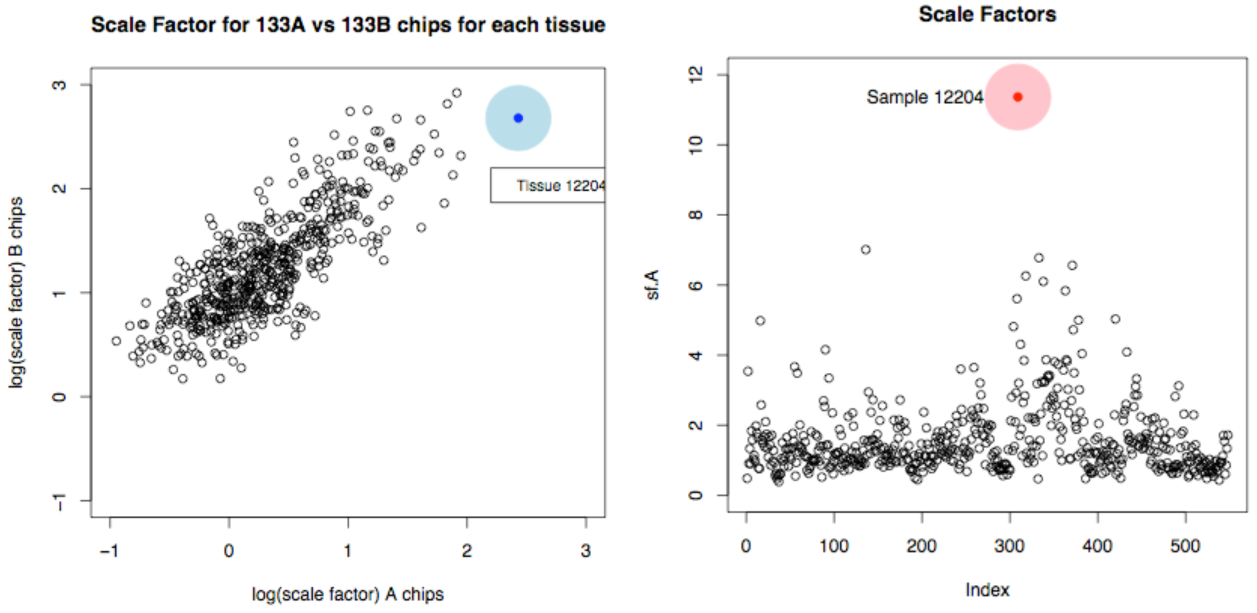

Figures 1A and 1B

Figure 1A shows the scaling factors for all arrays plotted for ChipA vs Chip B and Figure 1B shows the scaling values for A only. These data clearly suggest that the scaling factor applied to Tissue 12204 is exceptionally high for chip A and on the high range for Chip B **[Tissue 12204 will be removed from the 'scrubbed' data set].**

Background Values

According to Affymetrix quality guides, the background level should be similar across all chips[3]. Aberrantly high background levels for a particular array may indicate a problem with cRNA concentration, poor washing after hybridisation, or other experimental anomaly.

Figure 2 shows the background values for Chip A vs. Chip B for all arrays. Tissue 3424 clearly has exceptionally high background levels. **[Tissue 3424 will be removed from the 'scrubbed' data set.]**

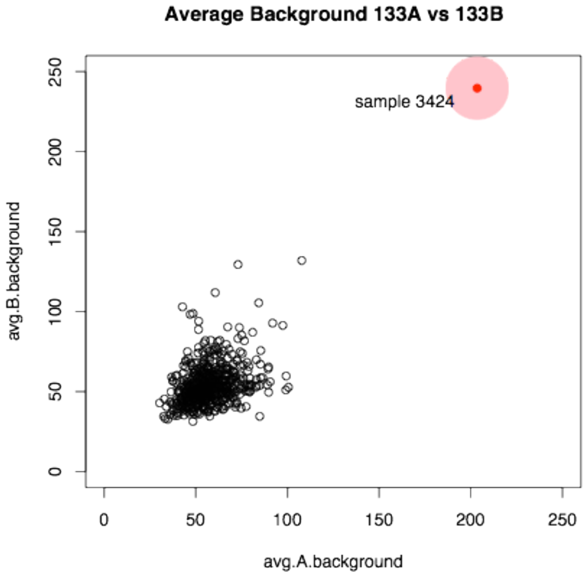

Figure 2

Percent Present

MAS5.0 detection calls (absent, present, marginal) are made for each gene based on the difference between perfect match (PM) and mismatch (MM) probes. [10] While this parameter may be misleading in terms of the absolute value of genes expressed, (as with other parameters) a wildly aberrant value for a particular chip may indicate unintended experimental variation.

Figure 3. shows a histogram/distribution of the percent of probesets called 'present' across all chips. Visual inspection of this graph does not suggest outliers. However, Figure 4 shows the percent present calls for the A chips plotted against the corresponding values on the B chips. Clearly, Tissue 31754 is dissimilar to the rest of the arrays. Fig 4 also demonstrates the utility of combining the A and B data for outlier detection. While the values for 31754 are not particularly anomalous for either chip singly, the overall 'shape' of the data suggests that Tissue 31754 behaves differently than the rest of the samples. **[Tissue 31754 will be removed from the 'scrubbed' data set.]**

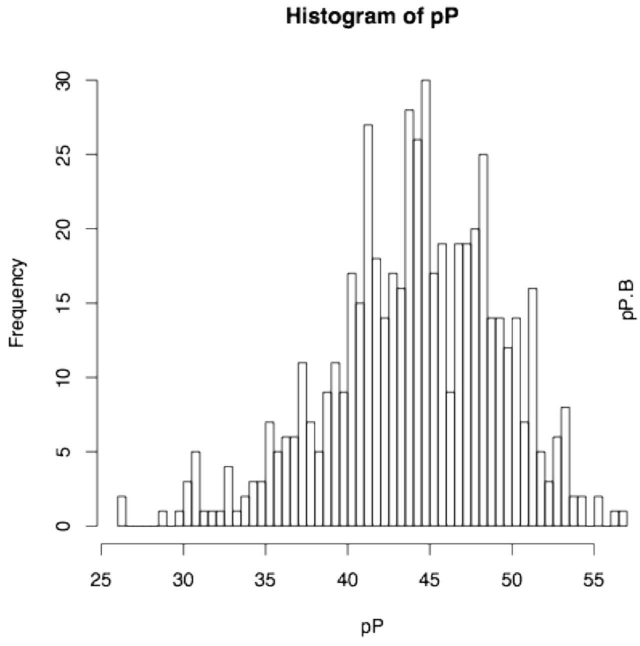

Figure 3

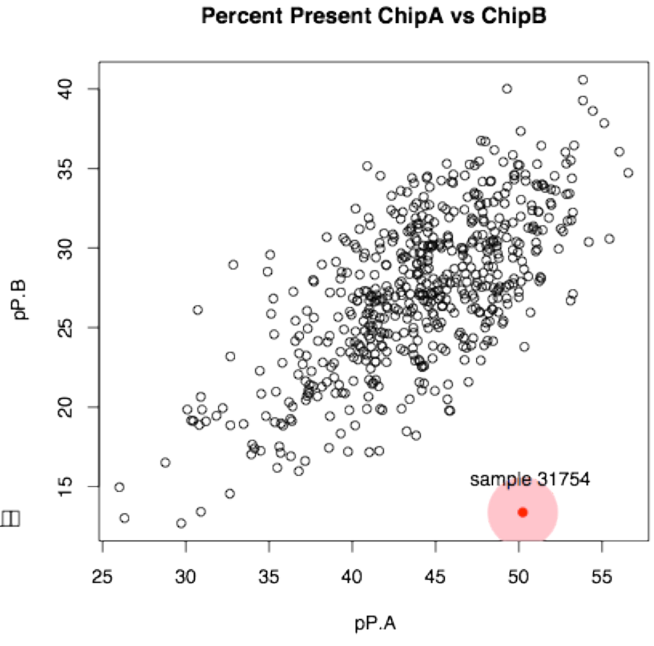

Figure 4

Spike-in probesets

According to standard Affymetrix Gene Chip protocols, *e. coli* transcripts BioB, BioC, BioD, and the P1 bacteriophage transcript CreX are spiked into the hyb solution at increasing concentration to confirm low-end assay sensitivity and appropriate dose response across the dilution range[3]. Figure 5. shows the probeset expression response across all 548 tissues. The observed response clearly does not match the expected linearly increasing expression values. Correspondence from Gene Logic has confirmed that the company does not spike in the bacterial control transcripts as per the Affymetrix guide. No quality assessment can be made from the spike-in controls.

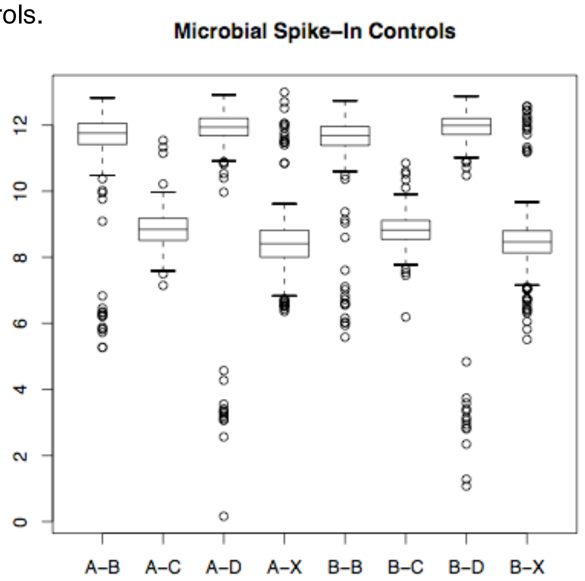

Figure 5

Control Probe Degradation

Affymetrix probeset sequences are generally chosen to react with approximately the last 600bp (3' terminus) of each gene or EST target transcript[11]. However, to test transcript efficiency and possible 5' degradation, two 'housekeeping' genes (GAPDH and β-actin) are each targeted at three locations along the entire gene transcript. For both of these gene targets, there is one probeset for each of the 3'-transcript tail, mid-transcript, and 5' -transcript head. By comparing the ratio of binding to the 3' tail against the binding to the mid- and 5' transcript, one may gain clues regarding sample transcript quality -- at least for these genes.

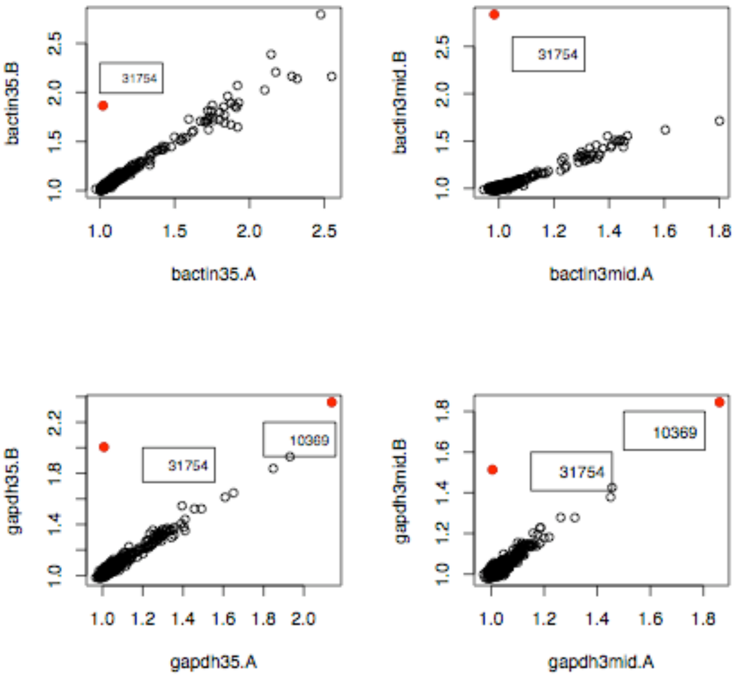

Figure 6

Figure 6 shows the β-actin and GAPDH ratios for 3':5' and 3':mid transcripts for chip A vs chip B.

Inspection of these data suggests that Tissue 31754 has a visibly different ratio profile across both of these genes and the ratios for 10369 are very high for both chip sets. A closer look at the 3'-mid ratio for GAPDH shown in Figure 7 further reveals that Tissue 10369 should conservatively be designated an outlier.

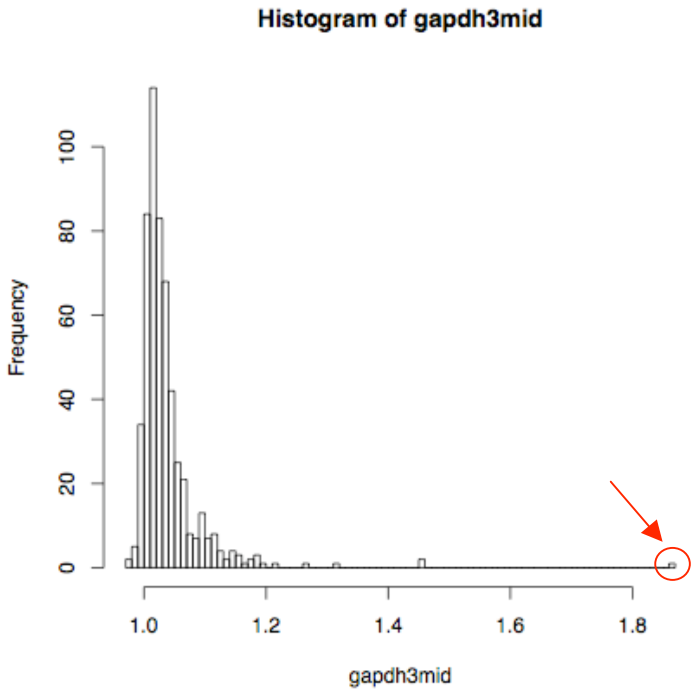

Figure 8

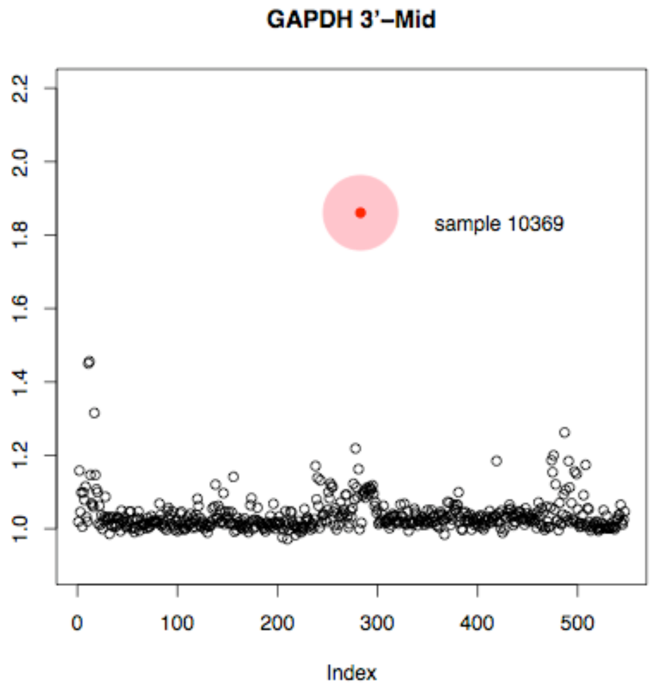

Figure 7

[Tissue 10369 will be removed from the 'scrubbed' data set. (Tissue 31754 has already been scrubbed.)]

RNA Degradation Analysis

In addition to Affymetrix hybridisation control data, Gene Logic has provided pre-reaction Bioanalyzer analysis of 28S:18S ratios for ribosomal RNA subunit intensities. The role of ribosomal RNA subunit ratios in the quality control process for microarrays is not clear and the literature is conflicting on their utility. Traditionally, a 28S:18S ratio of 2:1 has been an acceptable ratio for 'good' RNA and this ratio is suggested by Affymetrix. [11] However, these values are highly tissue variable and the ratio has been shown to be dependent on (for example) connective tissue levels, tissue RNase concentration, and whether or not the sample is tumour. [12] Several authors show that 28S:18S ratios can be misleading and find the ratio to be of 'no practical value.'[13] [14] More fundamentally, at least one author has concluded that these ratios are poorly indicative of the integrity or quality of the RNA sample. [14]

Gene Logic internal quality control procedures utilize a significantly lower threshold for this ratio, 0.5 or 1.0 (conflicting correspondence). [15] [16] Without access to the complete electrophoresis (or Bioanalyzer) chromatogram we explore 28S:18S values provided by Gene Logic and compare these to array qc probe (GAPDH, β-actin) results.

Note: Based on the data provided by GL on purchase, 148 tissues have missing values for the 28S:18S ratio.

Figure 9 shows the distribution of 28S:18S results across 400 arrays. Nearly all samples (99%) have ratio values less than the ideal 2:1 ratio and there is considerable variation about the mean (1.245, sd=0.325). There are three samples with ratio values greater than 2.25. While these three samples show discordantly high ratio results, we are not inclined to scrub such tissues without further information about specific peak profiles. Finally, the ratio distribution appears truncated at a lower minimum value of 0.5, suggesting that this is the lowest acceptable limit by GL.

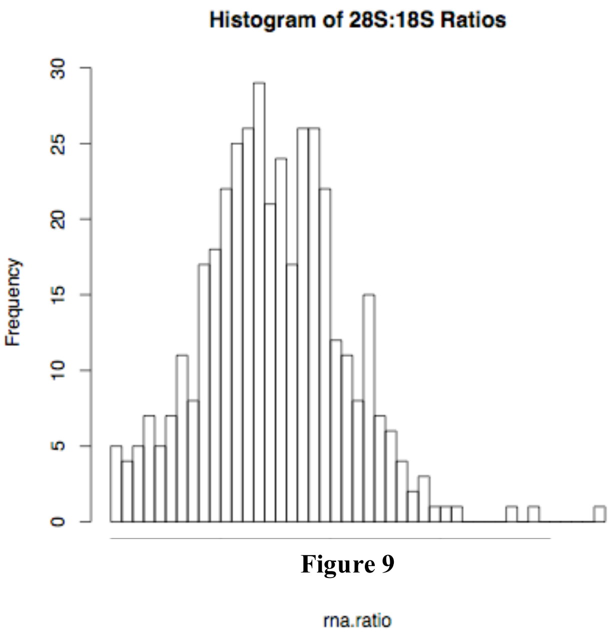

Figure 9

Within-probeset degradation

The final technique we use to explore potentially problematic tissues is to examine the total-array response for all 11-probe probesets (both PM and MM) across all genes. Generally, each transcript is targeted on the gene chip by 11 discrete (usually non-overlapping) perfect match (and mismatch) 25-mer probes. The mean intensity value for each of these individual probes provides information about the average binding response for all probesets on the chip. Thus, the first probe (#1) reacts with the 5' transcript target while the last probe (#11) reacts with the 3' transcript terminus.

Figure 10 shows the mean intensity for all chips at each probe location along the probeset. For illustration, this plot depicts only the first 20 arrays but the expected trend of a high intensity for the 3' probes relative to the 5' probe is readily apparent.

Another way to describe this binding trend is to calculate the positive slope for each array observed moving across the probesets (from 1 to 11 or, equivalently from 5' to 3'). Figure 11 shows the distribution of slope values across the A chips; the B chips yield a similar result, data not shown. Interestingly, these data suggest a bimodal distribution with a primary population slope near 2.0 and a secondary population with a higher value between 5.0 – 6.5. Further investigation suggests that most of these high-slope points correspond to a sub-population of arrays hybridised during 2004. This observation is potentially important because the majority of chips (503/548) were hybridised in 2002. See Table 1.

	2002	2003	2004
Arrays hybridised	503	17	28

Table 1

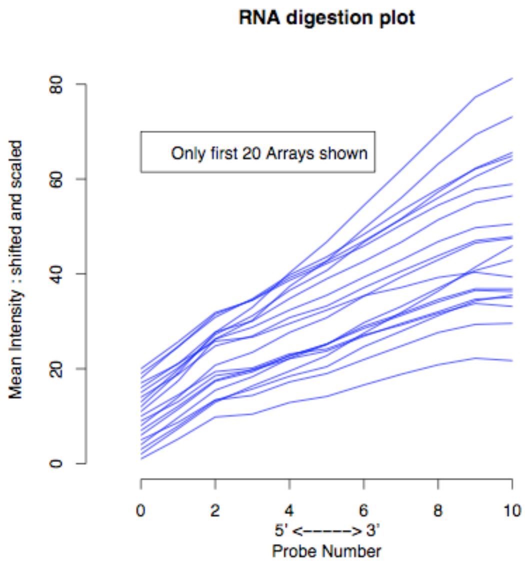

Figure 11

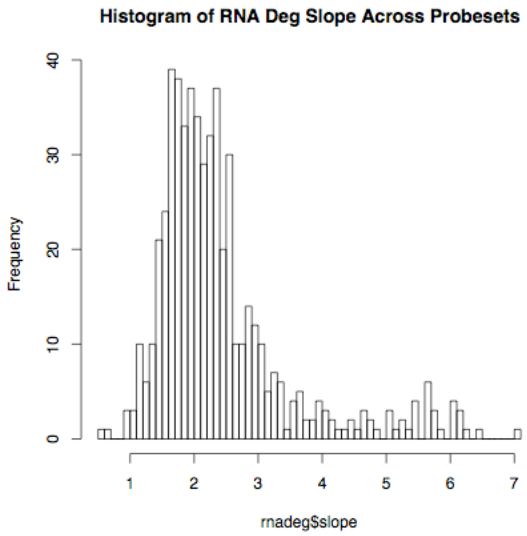

Figure 10

As with the other standard qc metrics analysed above, we can also explore the intra-tissue (or more correctly, the intra-hybridisation solution) response by plotting the A chip slope vs. the B chip slope (Figure 12). As usual, this technique of viewing intra-tissue response across both chips allows identification of possible outliers. **[This tissue (31754) was previously identified for removal from the scrubbed data set.]**

Figure 13 shows the same data (degradation slope chip A vs chip B) with highlighting for the 2003 and 2004 chips. As discussed above, we note that the '2004-hybridised' chips are disproportionately represented at the high end of the intra-probeset slopes. Subsequent investigation regarding the '2004' samples identified that these tissues were all processed using a 'Microsample Amplification' protocol which is applied to very small amounts of RNA, such as typically recovered in laser capture microarray techniques. Based on this new information these samples were REMOVED from our preliminary (conservative) analysis.

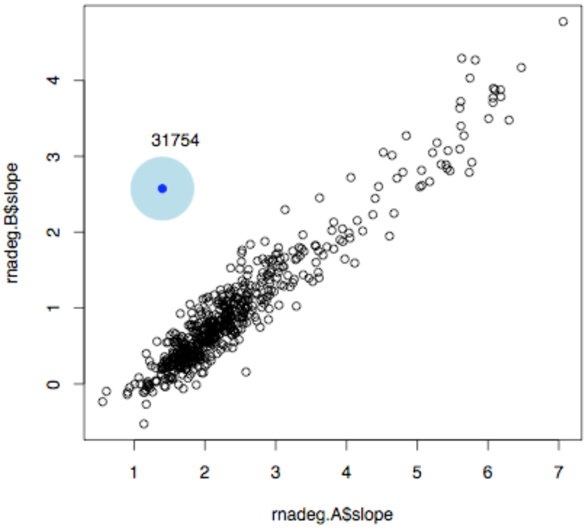

Figure 12

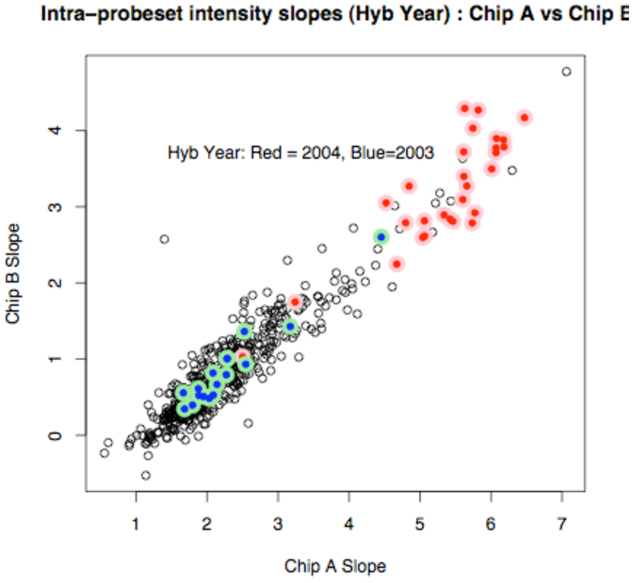

Figure 13

28S:18S Ratios

Finally, the intra-probeset binding slopes also allow us to revisit the question raised earlier regarding the utility of 28S:18S RNA subunit ratios to predict on-chip binding behavior. Our *a priori* expectation is that higher intra-probe set binding slopes should be observed for those tissues with relatively poor 28S:18S RNA ratios. Logically, we expect that tissues with an increased level of RNA degradation will yield lower binding for the 5' transcripts because there is less such product available due to preferential degradation of the 5' transcript. On the other hand, the 3' (with intact poly-A tail) will degrade more slowly and consequently yield a higher probe intensity. One might consequently expect that this (intrastrand) bias in the degradation process will result in arrays with higher slopes across the 11 25-oligomer probes. Figure 14 shows the intra-probeset slopes plotted against the 28S:18S ratio for the same tissues (recall that 148 tissues have missing ratio values).

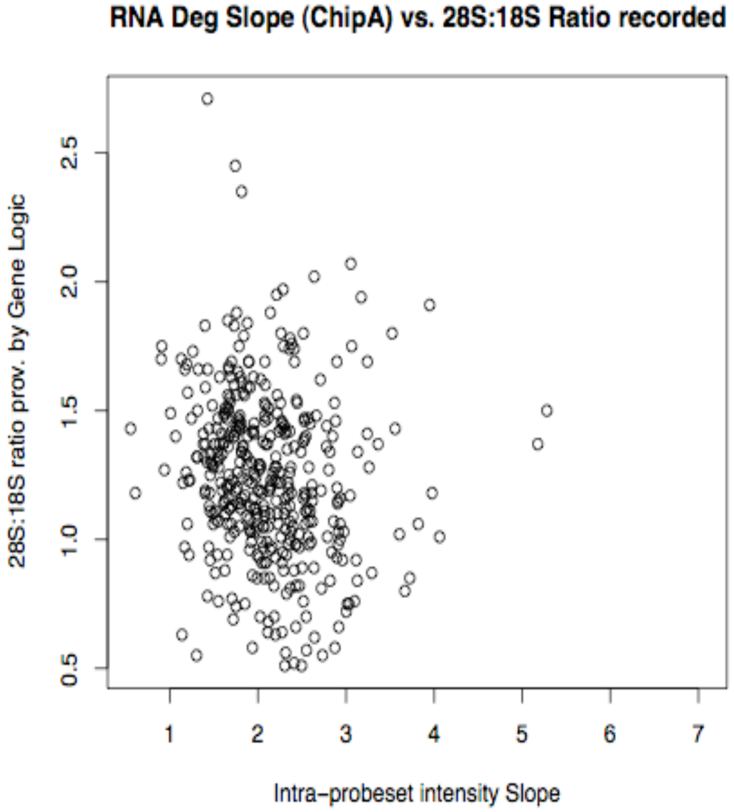

Figure 12

Visual inspection of Figure 14 suggests that there is marginal, if any, correlation between the value of 28S:18S ratio and the resulting intra-probeset ratio moving across the last 600 bases of each transcript. For example we note that the highest slope values (~5.0) shown here correspond to a relatively 'good' ribosomal subunit ratio (~1.5). Further the lowest ribosomal RNA ratios (~0.5) do not result in particularly high intra-probeset degradation slopes. These data support the conclusion that ribosomal subunit RNA ratios are poor predictors of on-chip binding behaviour.

Principal Component Analysis

Moving beyond the elementary quality analysis involved in outlier array detection, we also briefly explore the entire dataset using principal component analysis (PCA). This technique involves attempting to reduce the massively multivariate nature of the data matrix [n=548 samples, p=44,928 probesets (targets/genes)] to a new set of uncorrelated (orthogonal) variables that capture the essential variation structure of the data. By visually inspecting the data along the first several principal components, we seek to identify data-wide structure that may suggest fundemental variability within the data will need to be interpreted relative to experimental conditions. Such structure may be a warning that underlying experimental variation (by design or otherwise) could influence more sophisticated multivariate analysis.

Ultimately, the goal of PCA is to better understand the correlation structure within the data which may then suggest variable relationship hypotheses that can be further investigated. [2]

Figures 15 and 16 show the entire 548 arrays transformed into the first two principal components for the A chips and B chips, respectively.

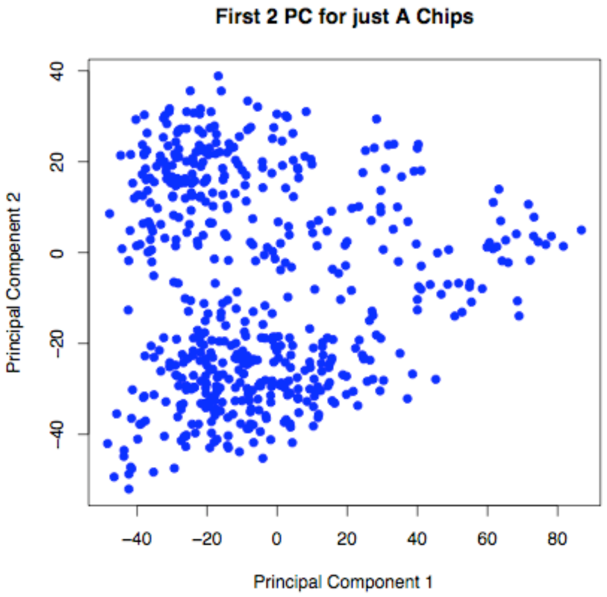

Figure 13

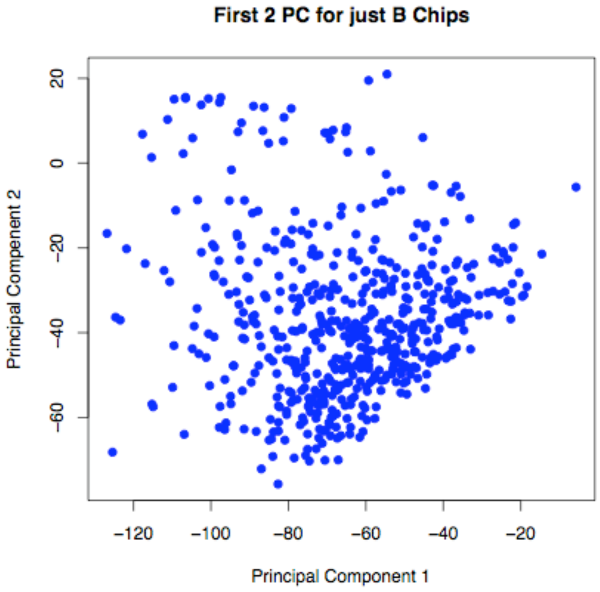

Figure 14

Visual inspection of these data hint that there may be two subpopulations of data within the 'A' chips delinated along the second component axis. The 'B' chips plot, on the hand, suggests a single diffuse data cloud in the first to component dimensions. This is interesting bearing in mind that the 'A' chip targets specific or hypothetical gene targets while the 'B' chip contains probesets intended to hybridise to less well defined expressed sequence tags (ESTs).

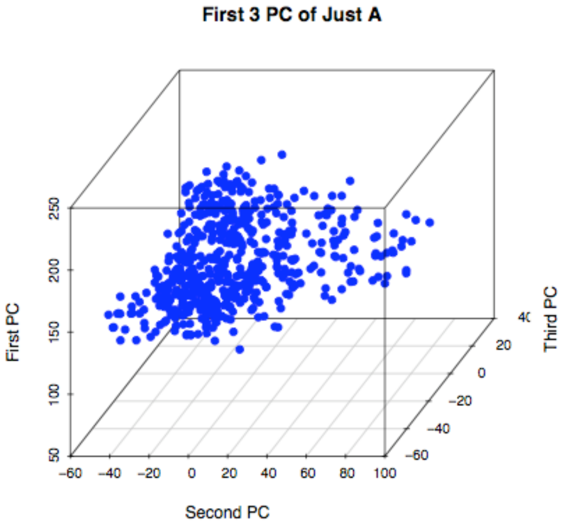

Figure 16

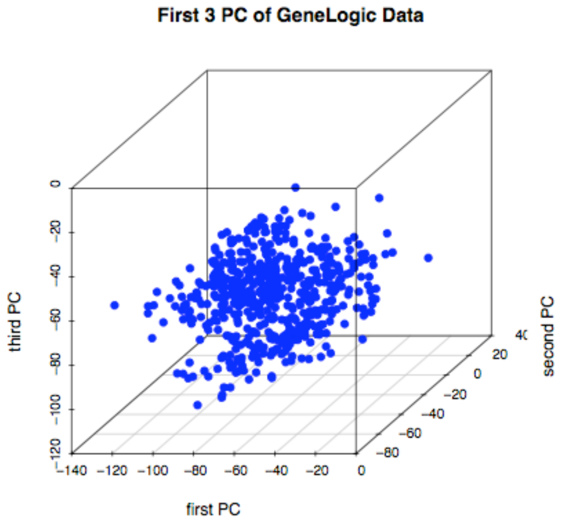

Figure 15

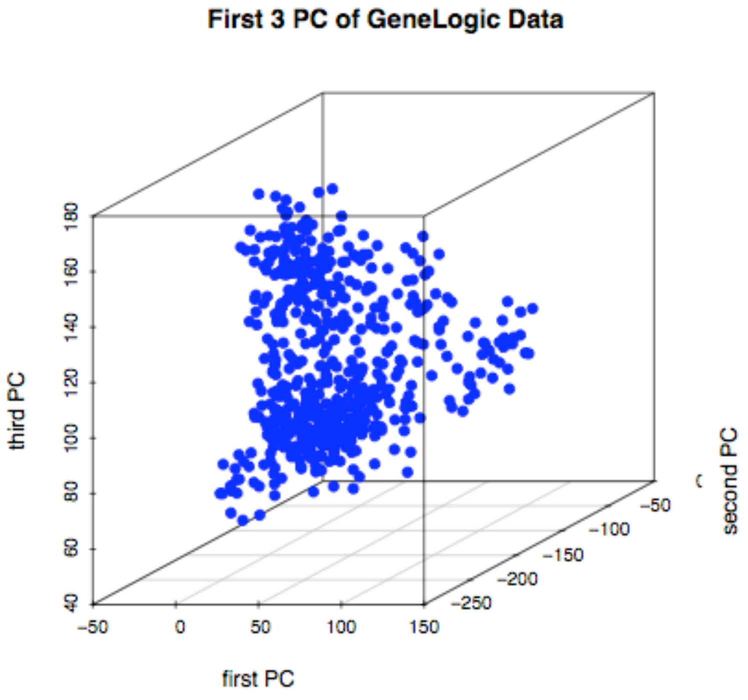

Figure 17

The data for chip A, chip B, and combined are shown in Figures 17, 18, and 19, respectively. The data structure evident in the two-dimensional view for the A chips is preserved in Figure 17 and also in the combined plot of Figure 19.

To attempt to identify experimental conditions that correlate with this bimodal observation, we display these data by repeatedly overlaying different highlight schemes based on experimental values treated as factors.

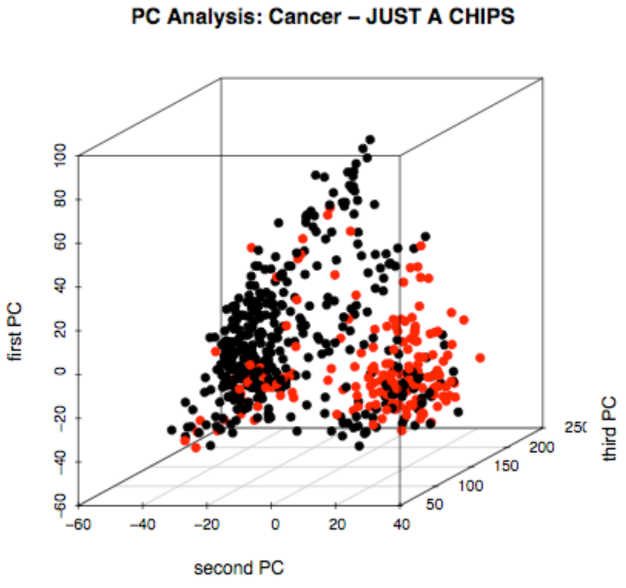

Figure 18

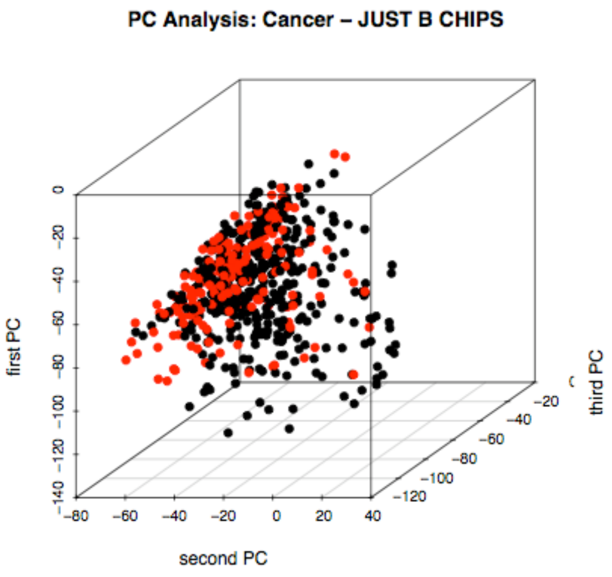

Figure 19

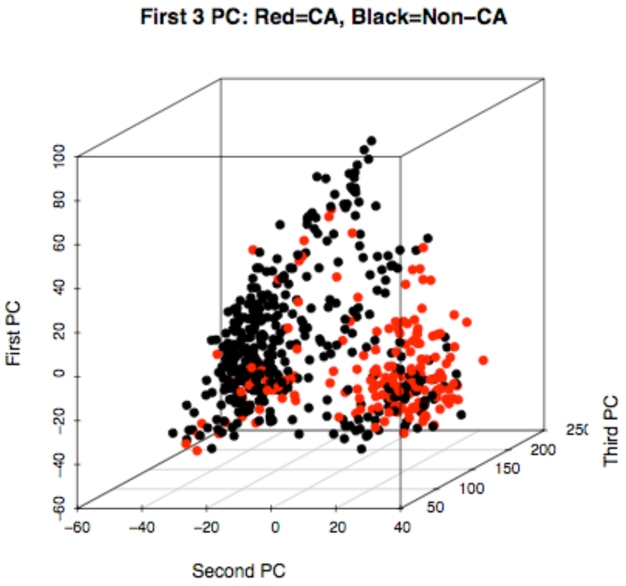

Figure 20

Visual inspection of the PCA plots with highlighting based on experimental factors was attempted for many possible variables, including: assay hybridisation date, operator, assay protocol, fluidics module, tissue donor gender, tissue donor race, age of tissue donor, sample tissue location within colon, and disease state (cancer vs. normal). Two dimensional plots are available as supplemental materials.

Interestingly, the only factor/variable that appears to correlate with the bimodal distribution is the tissue disease state as either cancer or normal. Plots for the first three components are shown for the A chips, B chips, combined in Figures 20, 21, and 22, respectively.

While this observation is very intriguing and will need to be investigated thoroughly, the PCA exploration did not identify any particular concerns that broad experimental factors have resulted in unexpected data behavior.

Scrubbed Array Summary

Based on this quality assessement and data review, we conclude that four tissues should be conservatively removed from the initial data mining experiments. Table 2 provides detail on these tissues.

Tissue Genomic ID	Location	Disease State	Comment
12204	Not given (colon)	Normal	normal colon taken from ileocolectomy for adenoma with severe atypia
3424	Ascending	Cancer	Adenocarcinoma of right colon with hepatic metastasis and node involvement
31754	Ascending	Normal	Normal right mucosa from hemicolectomy for carcinoid tumour of appendix
10369	Descending	Normal	Normal colon from left colectomy for adenocarcinoma
Multiple	Multiple	Multiple	All tissues processed using 'Microsample Amplification' (MSA) were also removed.

Code and Supplemental Material

All figures and R Scripts are available for secure execution (by authorised users only) on

```
ribosome://scratch/CRC15/genelogic/lcl/raw/qc_analysis/OUTLIER_ANALYSIS.R
ribosome://scratch/CRC15/genelogic/lcl/raw/qc_analysis/pca/PCA_ANALYSIS.R
```

For convenience, the final 'scrubbed' dataset resulting from this analysis has been RMA normalised and packaged. To load a normalised, clean version of the 'scrubbed' dataset, simply 'source' the following script:

```
ribosome://scratch/CRC15/genelogic/R/load_scrubbed_data.R
```

For further details regarding the clinical data provided by Gene Logic and a complete description of the data structures that are used for this analysis, see

```
ribosome://scratch/CRC15/genelogic/R/README.txt
```

References

1. Lipshutz RJ, Fodor SP, Gingeras TR, Lockhart DJ (1999) High density synthetic oligonucleotide arrays. Nat Genet 21: 20-24.
2. Chatfield, C., and Collins, A. J. (1981) Introduction to Multivariate Analysis (Chapman & Hall Statistics Text Series). Chapman & Hall/CRC.
3. Affymetrix (2001) Genechip Expression Analysis Data Analysis Fundamentals. Santa Clara, CA USA: Affymetrix Inc.
4. Wilson C, Miller CJ (2005) Simpleaffy: a BioConductor package for Affymetrix quality control and data analysis. Bioinformatics

5. Gautier L, Cope L, Bolstad BM, Irizarry RA (2004) affy--analysis of Affymetrix GeneChip data at the probe level. Bioinformatics 20: 307-315.
6. Gentleman RC, Carey VJ, Bates DM, Bolstad B, Dettling M, 0 (2004) Bioconductor: open software development for computational biology and bioinformatics. Genome Biol 5: R80.
7. Affymetrix (2001) Statistical Algorithms Reference Guide. Santa Clara, CA: Affymetrix Inc.
8. Hubbell E, Liu WM, Mei R (2002) Robust estimators for expression analysis. Bioinformatics 18: 1585-1592.
9. Irizarry RA, Bolstad BM, Collin F, Cope LM, Hobbs B, 0 (2003) Summaries of Affymetrix GeneChip probe level data. Nucleic Acids Res 31: e15.
10. Liu WM, Mei R, Di X, Ryder TB, Hubbell E, 0 (2002) Analysis of high density expression microarrays with signed-rank call algorithms. Bioinformatics 18: 1593-1599.
11. Affymetrix (2004) Gene Expression Analysis: Technical Manual. Santa Clara, CA: Affymetrix Inc.
12. Skrypina NA, Timofeeva AV, Khaspekov GL, Savochkina LP, Beabealashvilli RS (2003) Total RNA suitable for molecular biology analysis. J Biotechnol 105: 1-9.
13. Schoor O, Weinschenk T, Hennenlotter J, Corvin S, Stenzl A, 0 (2003) Moderate degradation does not preclude microarray analysis of small amounts of RNA. Biotechniques 35: 1192-6, 1198-201.
14. Dumur CI, Nasim S, Best AM, Archer KJ, Ladd AC, 0 (2004) Evaluation of quality-control criteria for microarray gene expression analysis. Clin Chem 50: 1994-2002.
15. GeneLogic (2005) Response to Biotechnology and Health Informatics: CSIRO Genomic Database Information.
16. GeneLogic (2005) Response to Biotechnology and Health Informatics CSIRO: Supplemental QC and Data Generation Questions.
